# Six-year positive effects of a mindfulness-based intervention on mindfulness, coping and well-being in medical and psychology students; Results from a randomized controlled trial

**DOI:** 10.1371/journal.pone.0196053

**Published:** 2018-04-24

**Authors:** Michael de Vibe, Ida Solhaug, Jan H. Rosenvinge, Reidar Tyssen, Adam Hanley, Eric Garland

**Affiliations:** 1 Norwegian Institute of Public Health, Oslo, Norway; 2 The Pain Clinic, University Hospital of Northern Norway, Tromsø, Norway; 3 Department of Psychology, Faculty of Health Sciences, The Arctic University of Norway, Tromsø, Norway; 4 Department of Behavioural Sciences in Medicine, Institute of Basic Medical Sciences, Faculty of Medicine, University of Oslo, Oslo, Norway; 5 College of Social Work, University of Utah, Salt Lake City, UT, United States of America; 6 Center on Mindfulness and Integrative Health Intervention Development, University of Utah, Salt Lake City, UT, United States of America; Brown University, UNITED STATES

## Abstract

Longitudinal research investigating the enduring impact of mindfulness training is scarce. This study investigates the six-year effects of a seven-week mindfulness-based course, by studying intervention effects in the trajectory of dispositional mindfulness and coping skills, and the association between those change trajectories and subjective well-being at six-year follow-up. 288 Norwegian medical and psychology students participated in a randomized controlled trial. 144 received a 15-hour mindfulness course over seven weeks in the second or third semester with booster sessions twice yearly, while the rest continued their normal study curricula. Outcomes were subjective well-being, and dispositional mindfulness and coping assessed using the Five Facet Mindfulness Questionnaire and the Ways of Coping Checklist. Analyses were performed for the intention-to-treat sample, using latent growth curve models. At six-year follow-up, students receiving mindfulness training reported increased well-being. Furthermore, they reported greater increases in the trajectory of dispositional mindfulness and problem-focused coping along with greater decreases in the trajectory of avoidance-focused coping. Increases in problem-focused coping predicted increases in well-being. These effects were found despite relatively low levels of adherence to formal mindfulness practice. The findings demonstrate the viability of mindfulness training in the promotion of well-being and adaptive coping, which could contribute to the quality of care given, and to the resilience and persistence of health care professionals.

**Trial registration:** Clinicaltrials.gov NCT00892138

## Introduction

Training in mindfulness, the deliberate awareness of moment-to-moment experience with an attitude of acceptance and non-judgement, is thought to foster health benefits and adaptive coping skills with potential lifelong relevance. However, little is known about effects occurring beyond one year after Mindfulness Based Interventions (MBIs), both in non-clinical [1–3], and clinical populations [4–6]. The current study seeks to fill this gap in knowledge by investigating six-year effects of mindfulness training in medical and psychology students using data from a multicenter RCT, and by exploring the positive impact of mindfulness training on well-being, coping skills and mindfulness disposition.

Medical and psychology students report high levels of mental distress and low levels of life satisfaction [7, 8]. As health care professionals, they are likely to continue reporting high levels of stress, burnout and suicide, relative to the general population [9–11]. Stress-related health problems are associated with patient dissatisfaction, worse patient outcomes, poorer patient safety and increased rates of professional errors [12, 13]. Sources of stress include high caseloads, high performance expectations, time pressure, treatment failure, dysfunctional organizational structure, ethical conflicts, and continuous exposure to human suffering [11, 14, 15]. Therefore, learning to cope with stress and exhaustion stands out as critical dimensions for health care professionals in training.

Coping strategies can be broadly divided into avoidance-focused, AFC (like ignoring, blaming, or avoiding) and problem-focused, PFC (like active problem-solving and positive cognitive reappraisal) strategies [16]. While AFC increases the risks for negative health outcomes when used inflexibly [17], problem-based strategies increase goal achievement, and is associated with a number of positive health outcomes [18, 19]. The time point at which these two coping strategies are deployed in response to a specific stressor is believed to contribute to their divergent trajectories [20]. PFC, exemplified by strategies like reappraisal, is thought to represent a more immediate, proactive coping strategy, thereby reducing the momentum of the stress response and potentially even shifting that momentum in more constructive directions [20]. In contrast, AFC can be understood as more reactive, a late-emerging strategy that may rely on cognitive and emotional suppression–techniques which may actually increase the sympathetically-mediated stress response over time [20].

Among university students, AFC has been associated with higher levels of perceived stress [21], lower satisfaction with life [22], and increased postgraduate mental health problems [23]. On the other hand, PFC strategies (such as active problem-solving), have been associated with fewer depressive symptoms [24].

MBIs have received increased interest in the field of health care education due to a growing body of research documenting enhanced psychological functioning following mindfulness training, both in clinical and non-clinical populations [2, 3, 25–27]. Yet, we lack long-term prospective studies on possible mechanisms and factors that promote healthy effects. MBIs aim to cultivate a present-oriented and non-judging quality of attention, achieved through various meditative practices. The practice of repeatedly attending to moment-to-moment experience while letting go of distracting thoughts and emotions, is thought to foster a decentered perspective from which habitual automatic cognitions and emotional reactions can be viewed from a “psychological distance”, promoting affect tolerance, cognitive flexibility, and reduced worry and rumination [28–30]. From such a decentered, broadened state of awareness, it is theorized that productive reengagement with stressful life events may be facilitated [31]. Hence, mindfulness training may promote PFC while reducing AFC strategies.

Evidence for the link between mindfulness and adaptive coping is emerging [32–34]. However, surprisingly few studies have addressed the effect of mindfulness training on coping in health care professionals or trainees, and long-term follow-up studies are lacking. A few previous MBI studies of other population samples have shown enhanced coping skills, i.e. predominantly reduced avoidance coping in clinical populations [35, 36], healthy adults [33, 37], and college students [38]. However, only two of these studies used RCT designs [33, 36]. Post-intervention data from the current trial found increased use of PFC following the MBI, and students, high on neuroticism scores, showed reduced AFC [39]. These changes were maintained at two and four-year follow-up [40].

The present study uses follow-up data from the same trial to examine the effect of an MBI on changes in dispositional mindfulness, coping style (AFC, PFC, and seeking social-support coping, SS), and well-being over a six-year period. This has never been done before, and will allow us to see whether the effects of a mindfulness intervention can hold up over time, and to examine if changes in mindfulness and coping strategies can account for long-term effects on well-being.

## Materials and methods

### Participants and procedures

**Fig 1:**
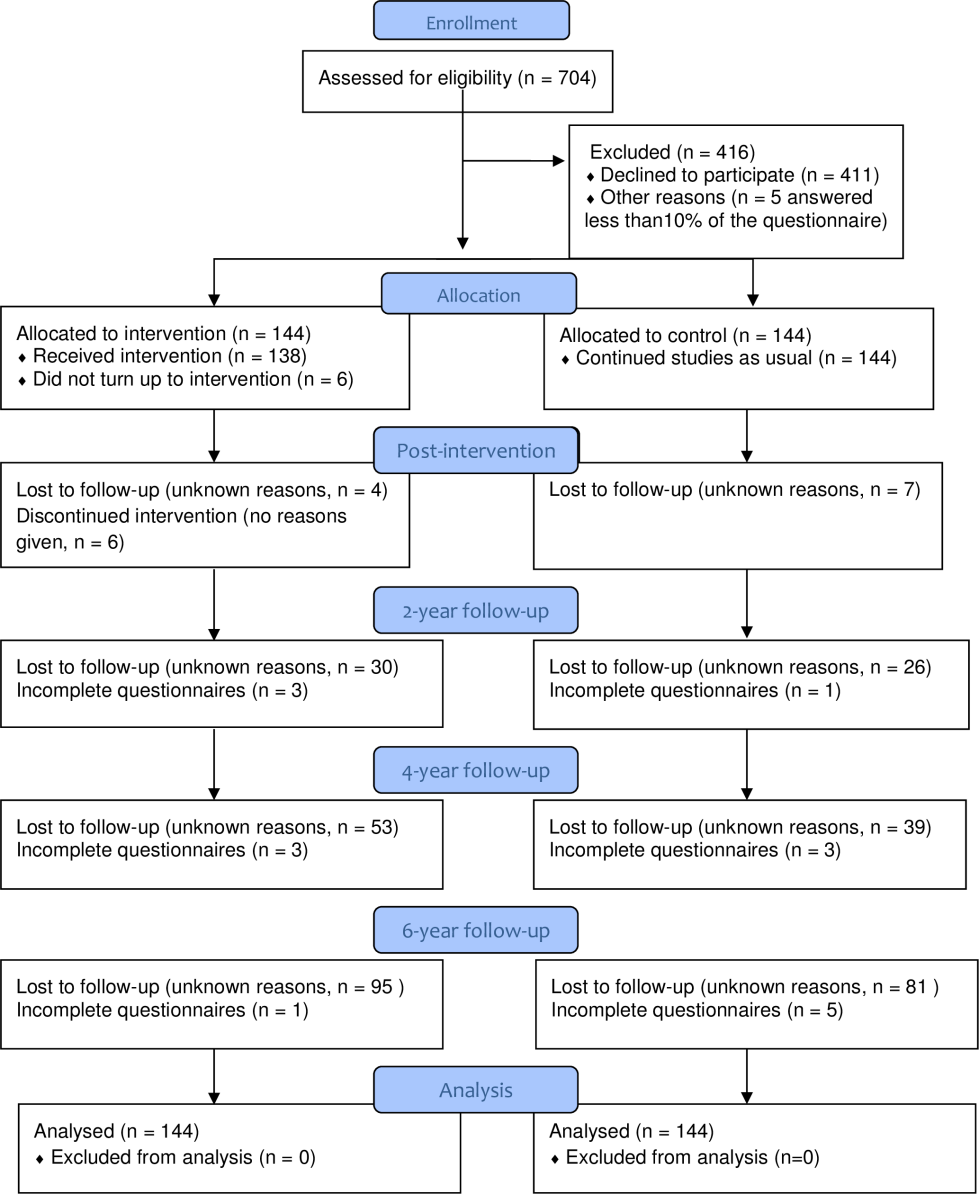
Flow diagram.

Of 704 eligible first -and second-year medical and clinical psychology students from two Norwegian universities, 288 (mean age: 24 years; 219 women; 176 medical students and 112 psychology students) participated in the study during 2009 and 2010, with 144 allocated to the intervention and 144 to continue their studies as scheduled. The sample size was calculated based on longitudinal studies of how study stress and mental distress increases over the study curriculum in Norwegian medical students (10). In the power calculation we assumed that the intervention would prevent this increase. With an alpha level of .05 and 80% power, 60 to 100 participants were needed in each study group. The investigators were blinded to group allocation, as a technician, not otherwise involved in the study, ran a computer randomisation programme and concealed allocation until baseline measurements had been collected. He also administered the online website for filling in the questionnaires and kept the code that identified the students. Apart from baseline, data were collected one month and two, four and six years after the intervention. At each data collection, students received a book voucher of $50 for their participation. Details regarding the procedure and baseline characteristics are described elsewhere [41]. Fig 1 shows the flow diagram for the study.

All participants provided informed consent. The study was approved by the Regional Committee for Medical and Health Research Ethics in Norway and the Norwegian Data Inspectorate. The Study Protocol is registered on Clinicaltrials.gov (NCT00892138), see S1 Protocol.

### Programme description

Students in the intervention group participated in a 7-week abridged Mindfulness-Based Stress Programme (MBSR). This was similar to that developed by Kabat-Zinn [42], but shorter in duration (reduced from 8 to 7 weeks), and less intense (reduced from 2.5-hr sessions to 1.5-hr sessions and from 45 to 20–30 min of recommended home-based mindfulness practice), with a full day of mindfulness practice retained at week seven. The intervention is described in detail elsewhere [41]. During the six-year follow-up period, students in the intervention group were invited to participate in optional 1.5-hour mindfulness booster sessions once every semester.

### Measures

Well-being was measured using four items [43]. In accordance with consensus regarding essential well-being dimensions [44], the well-being assessment included items measuring cognitive life satisfaction, positive affect (happy and strong), and negative affect (unhappy and tired). As these items used different response categories, all were transformed to a scale ranging from 0 to 10, using the following formula: X = (Y—1) × 10 / (Z—1), where Y and Z represent the original scores and number of response categories, respectively. Higher scores reflect increased well-being. The scale has demonstrated good psychometric properties, and construct validity was indicated via strong correlation with the Satisfaction With Life Scale in a student sample [44], and a Cronbach’s α of .81 in the present sample.

Coping was measured using the 42-item Ways of Coping Checklist, which contains five coping dimensions ‘problem-focused coping’, ‘seeking social support’, ‘self-blaming’, ‘avoidance’, and ‘wishful thinking’ [45]. Problems in replicating the original factor structure [22, 46], prompted the use of principal components factor analysis for our dataset, see S1 Text. This analysis justified retaining only three components. The first, ‘problem-focused coping’ (PFC) (α = .79), consisted of 14 items related to cognitive coping (i.e. identifying new ways of looking at the situation and benefit finding) and active problem solving. The second, ‘avoidance-focused coping’ (AFC) (α = .82), comprised 17 items related to blaming oneself, wishful thinking, and avoidance. The third, ‘seeking social support’ (SS) (α = .86), consisted of nine items related to seeking help and advice, including three reverse-scored items pertaining to hiding one’s feelings and avoiding social contact. Higher scores represent increased use of the coping strategies.

Dispositional, or trait mindfulness, was measured using the 39-item Five Facet Mindfulness Questionnaire (FFMQ), in which four facets contain eight items each, and one facet contains seven items. The five response categories range from 1 (never or very seldom true) to 5 (very often or always true). Higher scores indicate increased mindfulness. The psychometric properties of the scale are good [47], and the questionnaire has been validated in a Norwegian student population [48]. The facets and their corresponding Cronbach’s αs in the current study were as follows: ‘observing’ (.78), ‘describing’ (.89), ‘acting with awareness’ (.88), ‘non-judging of inner experience’ (.92), and ‘non-reactivity to inner experience’ (.73).

Student compliance was measured according to class attendance and the extent of home-based mindfulness practice. Attendance was represented by the number of classes attended (0–7). Two questions were used to measure the frequency (six categories, with responses provided using a scale ranging from 0 [never] to 5 [daily]) and duration (six categories, with responses provided using a scale ranging from 0 [0 min] to 5 [>45 min]) of formal mindfulness home practice during the preceding four weeks.

### Statistical analysis

An Independent sample T-test was used to determine whether participants randomized to the mindfulness condition differed significantly from control participants in their baseline levels of DM, AFC, PFC, SS, and well-being. An additional T-test was used to determine if between group differences existed in the primary variables at baseline for study completers and non-completers. Frequency counts were used to characterize intervention compliance and mindfulness practice involvement across the course of the study. Pearson correlation analysis was used to explore bivariate correlations between the primary variables of interest at each measurement point.

Five latent growth curve (LGC) models were constructed to examine the effect of treatment related changes in DM, AFC, PFC and SS on well-being. First, *four univariate* LGC models were fitted for the repeated DM, AFC, PFC and SS measures to assess each constructs’ temporal stability and change. For each construct a latent intercept factor, indicative of the measured construct’s stable, trait-like qualities, and a latent slope factor, indicative of the measured construct’s rate of change was derived. The intercept factor was estimated by fixing the factor loadings for all repeated measures to 1. The slope factor was estimated by setting the factor loadings of each successive time point to 0, 1, 2, 4, and 6, respectively, to reflect the time differences between each assessment point. The influence of mindfulness training on the trajectory of the four normally distributed, homoscedastic, independent variables of interest (DM, AFC, PFC, and SS) was explored by regressing each univariate LGC model on a variable representing treatment condition (MBI vs. Control) to investigate between-group differences in the trajectory of the modelled variable over time. Additionally, a residualized change score for well-being (Time 6 residualized on Time 0, representing well-being at six-year follow-up controlling for baseline differences in well-being) was positioned as the outcome variable in each univariate model to evaluate the effect of treatment related change trajectories in the modelled variable on well-being.

Second, a multivariate LGC model was fitted for a model combining each construct evidencing a treatment effect. This multivariate LGC model was also regressed on a variable representing treatment condition. Residualized change in well-being was again positioned as the outcome variable in the multivariate LGC model to evaluate the effect of treatment related changes in the trajectory of each treatment sensitive variable on well-being. Amos 24 was used to construct all LGC models. Full Information Maximum Likelihood Estimation procedures were used to handle missing data. Model fit was evaluated using the relative chi-square ratio (X2/df), comparative fit index (CFI) and root mean squared error of approximation (RMSEA). A model with a chi-square ratio below 3 [49, 50], CFI above .95, and RMSEA below .06 is believed to demonstrate good fit [51].

## Results

### Descriptive analyses

Descriptive statistics for baseline data are shown in Table 1. With the exception of gender, where significantly more men were allocated to the control group (*N* = 43 vs. 26), the randomisation procedure was effective, as no outcome measures or demographic variables showed a significant group difference.

**Table 1:** Socio-demographic characteristics for the intervention and control groups at baseline.

Characteristic	Overall	Intervention	Control	
	*N* = 288	*n* = 144	*n* = 144	*p v*alue
Mean age (*SD*)	23.8 (5.2)	23.6 (4.7)	24 (5.7)	.58
Women, *n* (%)	219 (76)	118 (82)	101 (70)	.03
Site, *n* (%)				.63
Oslo	179 (62)	87 (60)	92 (64)	
Tromsø	109 (38)	57 (40)	52 (36)	
Study field, *n* (%)				.72
Medicine	176 (61)	86 (60)	90 (62)	
Psychology	112 (39)	58 (40)	54 (38)	
Relationship status, *n* (%)				.16
Married/cohabiting	86 (30)	37(26)	49 (34)	
Single	202 (70)	107 (74)	95(66)	
No. of children, *n* (%)				.34
0	269 (93)	137 (95)	132(92)	
1–5	19 (7)	7 (5)	12 (8)	

#### Study flow, attrition, and comparisons between completers and non-completers

Fig 1 illustrates the study flow diagram. Dropout rates for T1, T2, and T4 and T6 were 3%, 19%, 32%, and 61%, respectively. Study completers were observed to have lower levels of baseline avoidance coping than non-completers: t(285) = 2.83, p = .005. Apart from that, baseline outcome measures did not differ significantly between dropouts and completers. Data were found to be missing at random (*X*^2^ = 218, df = 228, p = .67).

#### Intervention compliance and mindfulness practice

Students attended an average of 5.3 MBSR sessions (*SD* 1.9, range 1–7). During the six-year follow-up period, 21% attended one booster session, 25% attended between two and four sessions, and 5% attended between five and eight sessions, while 46% of students declined to join the eight booster sessions. Booster-session attendance did not predict variation in any of the outcome measures.

Details regarding home mindfulness practice frequency and duration during the four-year follow-up period can be found in Solhaug 2017 [40]. During the six-year follow-up period, the number of participants in the intervention group who reported to practice formal mindfulness exercises decreased from 112 of 140 (80%), one month after the intervention to 28 of 48 (58%), at six-year follow-up, while it increased in the control group from 35 of 138 (25%) to 21 of 58 (36%) participants. At 6-year follow-up, the students in both the intervention and control group who reported to practice, did so approximately once a week for 15 minutes. The number of control group participants that reported to have attended a course in qigong, yoga, tai chi, relaxation, or meditation) during the 2, -4-, and 6-year follow-up periods were 26 of 116, 27 of 102, and 7 of 58, respectively.

#### Univariate statistics / correlations between mindfulness and coping outcomes

Table 2 reports means, standard deviations and correlations for the each of the variables of interest at the relevant time points.

**Table 2:** Correlations and univariate statistics for dispositional mindfulness, problem-focused coping and avoidance-focused coping in the intervention group, and means and standard deviations for intervention and control group over five time points.

	Dispositional Mindfulness					Problem-Focused Coping					Avoidance-Focused Coping					Social-Support Coping					Well-Being	
Variable	DM 0	DM 1	DM 2	DM 4	DM 6	PFC 0	PFC 1	PFC 2	PFC 4	PFC 6	AFC 0	AFC 1	AFC 2	AFC 4	AFC 6	SS 0	SS 1	SS 2	SS 4	SS 6	WB 0	WB 6
DM 0	-																					
DM 1	.68***	-																				
DM 2	.60***	.73***	-																			
DM 4	.55***	.70***	.65***	-																		
DM 6	.57***	.65***	.63***	.80***	-																	
PFC 0	.52***	.43***	.33***	.45***	.18	-																
PFC 1	.39***	.57***	.37***	.46***	.10	.67***	-															
PFC 2	.41***	.48***	.53***	.48***	.28	.46***	.62***	-														
PFC 4	.42***	.49***	.48***	.70***	.37*	.52***	.63***	.65***	-													
PFC 6	.29*	.35*	.41**	.57***	.37**	.36*	.58***	.64***	.76***	-												
AFC 0	-.54***	-.40***	-.39***	-.52***	-.53***	-.36***	-.16	-.30**	-.34**	-.21	-											
AFC 1	-.39***	-.49***	-.45***	-.55***	-.47**	-.26**	-.25**	-.29**	-.37***	-.23	.74***	-										
AFC 2	-.38***	-.42***	-.59***	-.57***	-.49**	-.29**	-.18	-.39***	-.37**	-.36*	.69***	.74***	-									
AFC 4	-.40***	-.47***	-.45***	-.67***	-.62***	-.17	-.17	-.27*	-.37***	-.27	.64***	.69***	.72***	-								
AFC 6	-.39**	-.37*	-.38*	-.58***	-.67***	-.07	.06	-.25	-.17	-.30*	.69***	.70***	.63***	.84***	-							
SS 0	.33***	.23*	.20*	.38***	.10	.31***	.33***	.26**	.27*	.18	-.39***	-.33***	-.28**	-.33**	-.08	-						
SS 1	.28**	.34***	.31**	.41***	.20	.29**	.39***	.31**	.29**	.24	-.29***	-.36***	-.29**	-.36**	-.19	.79***	-					
SS 2	.25*	.21*	.32**	.37**	.14	.24*	.22*	.35***	.34***	.27	-.27**	-.29**	-.34***	-.30**	-.04	.69***	.73***	-				
SS 4	.23*	.27*	.28**	.56***	.24	.18	.21*	.35**	.47***	.24	-.39***	-.34**	-.39***	-.50***	-.35*	.61***	.59***	.72***	-			
SS 6	.17	.18	.29	.40**	.25	-.12	.02	.15	.24	.19	-.28	-.22	-.09	-.35*	-.29*	.51***	.60***	.59***	.80***	-		
WB 0	.54***	.33***	.24*	.35**	.17	.53***	.32***	.19*	.21*	.11	-.51***	-.39***	-.31**	-.22*	-.18	.34***	.24**	.18	.15	.01	-	
WB 6	.49***	.45**	.33*	.60***	.56***	.36*	.26	.29*	.19	.39***	-.45***	-.32***	-.29	-.39**	-.49***	.28	.25	.11	.20	.16	.46**	-
Intervention Mean	125.11	130.28	132.79	136.30	140.53	34.56	36.39	37.22	37.10	36.58	32.76	30.52	28.08	26.91	22.67	21.65	22.89	22.99	23.48	24.83	6.31	6.95
S.D.	16.35	16.17	17.07	16.33	15.28	6.04	6.11	5.83	6.02	4.81	9.66	10.01	9.23	10.60	9.07	6.07	5.84	6.20	6.00	5.35	1.75	1.43
Control Mean	127.10	127.77	131.08	132.41	134.28	35.62	35.35	36.00	35.08	34.84	31.84	31.18	28.79	28.41	27.07	22.27	22.34	22.91	23.83	23.67	6.41	6.30
S.D.	14.45	15.45	16.80	18.47	15.72	6.41	6.14	6.76	6.40	6.80	9.16	9.56	9.29	9.81	8.58	5.82	5.68	5.13	5.32	5.41	1.76	1.79

DM = Dispositional Mindfulness, PFC = Problem-Focused Coping, AFC = Avoidance-Focused Coping, SS = Social-Support Coping, WB = Well-Being, S.D. = Standard Deviation. 0 = baseline, 1 = 1 month after intervention, 2, 4 and 6 = 2, 4 and 6 years after the intervention

*p < .05

**p < .01

***p < .001

Generally, correlation strengths ranged from small (0.3) to medium (0.6), with most correlations demonstrating moderate magnitudes of association. Correlations among DM scores across each time point were high, suggesting stability in DM. Correlations among all three coping style scores were similarly high, also suggesting construct stability. Associations between the three constructs at similar time points demonstrated stronger correlations.

### The influence of a MBI on trajectories of change and well-being

The influence of mindfulness training on the trajectory of the four variables of interest (DM, AFC, PFC and SS) was explored by regressing each univariate LGC model on a single, dichotomous treatment condition item (MBI vs. Control). The DM (*X*^2^ = 24.70, *df* = 13, *p* = .03, *X*^2^/*df* = 1.90, CFI = .98, RMSEA = .06), AFC (*X*^2^ = 27.84, *df* = 13, *p* = .01, *X*^2^/*df* = 2.14, CFI = .98, RMSEA = .06), and PFC (*X*^2^ = 25.34, *df* = 13, *p* = .02, *X*^2^/*df* = 1.95, CFI = .97, RMSEA = .06), all evidenced good fit, with treatment condition emerging as a significant predictor of each construct’s slope (*β* = .18, *p* = .04; *β* = -.23, *p* = .01; *β* = .40, *p* < .001, respectively). The SS model evidenced poorer fit (*X*^2^ = 49.75, *df* = 13, *p* < .001, *X*^2^/*df* = 3.827 CFI = .92, RMSEA = .10), and treatment condition was not found to predict changes in SS (*p* = .17). As such, SS was not included in the multivariate LGC model.

**Fig 2:**
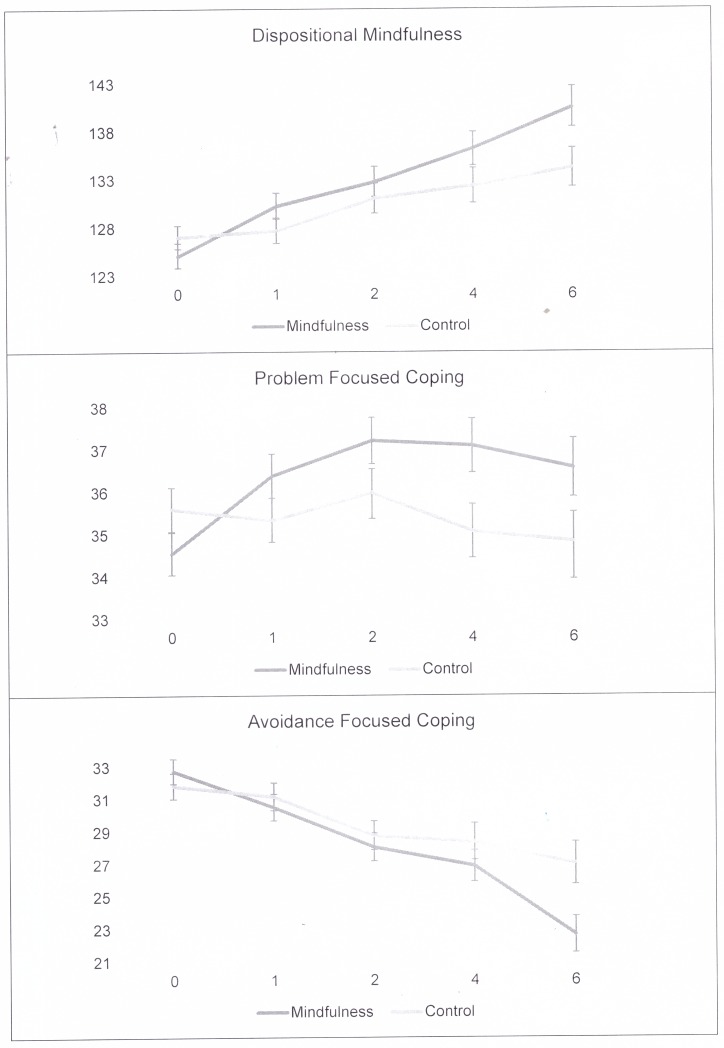
Line graphs depicting mean values and standard errors for each treatment sensitive variable at each measurement point.

The influence of mindfulness training on the trajectory of DM, AFC, and PFC over the 6-year period was examined by regressing a multivariate LGC model on treatment condition. Line graphs for each treatment sensitive variable is shown in Fig 2.

In addition, a residualized change score for well-being was regressed on the latent intercept and slope factors of DM and both coping styles. In the initial model, inter-correlations were added between the latent slopes and intercepts of DM, AFC, and PFC. This model evidenced sub-optimal fit: *X*^2^ = 356.50, *df* = 121, *p* < .001, *X*^2^/*df* = 2.95 CFI = .90, RMSEA = .08 (*CI* = 0.07, 0.09). Within-year correlations were then added between DM, AFC and PFC at each model time point. Adding these correlations resulted in significantly improved model fit (*X*^*2*^ *change* = 199.22, *df change* = 15, *p* < .001), and a final model that fit the observed data very well: *X*^2^ = 157.28, *df* = 106, *p* = .001, *X*^2^/*df* = 1.48 CFI = .98, RMSEA = .04 (*CI* = 0.03, 0.05).

**Fig 3:**
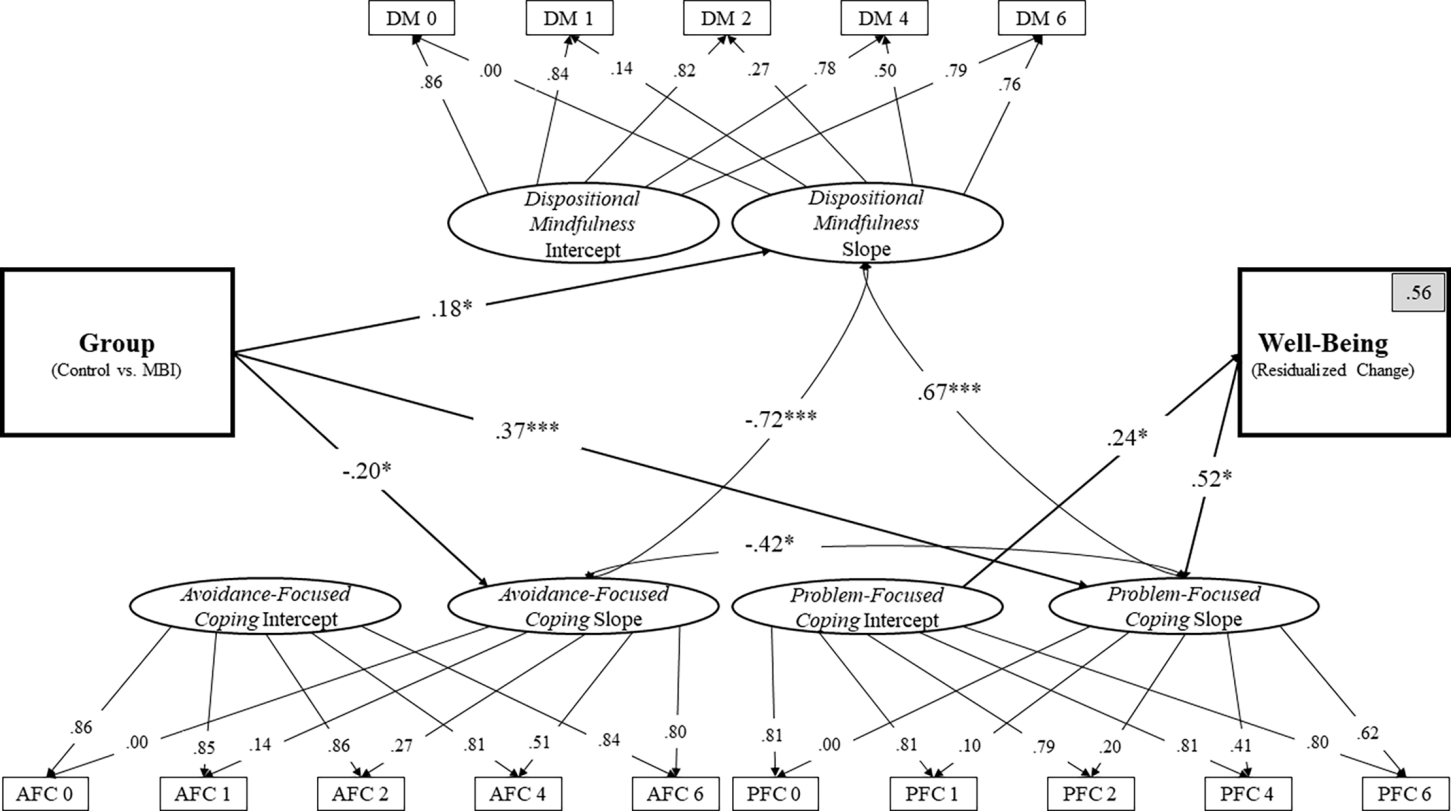
Final latent growth curve model of the influence of a MBI on dispositional mindfulness, avoidance-focused coping and problem-focused coping in relation to well-being. Note: Non-significant paths are not depicted. DM = Dispositional Mindfulness. AFC = Avoidance-Focused Coping. PFC = Problem-Focused Coping. The gray box in the top right corner of the well-being variable indicates the percentage of variance accounted for in well-being by the entire model. *p < .05, **p < .01, ***p < .001.

In this final model (Fig 3), each construct evidenced a significant slope—DM (*p* < .001), AFC (*p* = .02), and PFC (*p* < .001)—suggesting significant increases in DM and PFC along with significant decreases in AFC over the six-year period. Significant negative correlations were observed between the slopes of AFC and DM (*r* = -.72, *p* < .001), as well as between the slopes of AFC and PFC (*r* = -.42, *p* = .03). Significant positive correlations were observed between the slopes of DM and PFC (*r* = .67, *p* < .001). Taken together, these results indicate that increases in DM were associated with increases in PFC and that increases in both DM and PFC were associated with decreases in AFC over the observed six-year time period.

A significant effect was identified for treatment condition on the latent slope factors for DM (*β* = .18, *p* = .04), AFC (*β* = -.20, *p* = .02), and PFC (*β* = .38, *p* < .001), suggesting that relative to the control group, the MBI group exhibited significantly greater increases in DM and more problem-focused coping styles over time. Treatment condition was not significantly associated with any of the three constructs’ latent intercept factor, indicating that treatment groups did not differ at baseline (Time 0). With respect to residualized change in well-being, PFC was found to be the strongest predictor. The PFC intercept (*β* = .24, *p* = .04) and slope (*β* = .52, *p* = .02) factors were both significant predictors of the residualized change score in well-being. Neither the DM intercept (*β* = .07, *p* = .66) or slope (*β* = .15, *p* = .63) factors, or the AFC intercept (*β* = -.03, *p* = .84) or slope (*β* = -.04, *p* = .86) factors were found to predict well-being. The full model accounted for 56% of the variance in well-being at the six-year time point after controlling for well-being at baseline.

Sensitivity analyses revealed that including group attendance or amount of home mindfulness practice as predictors in the final multivariate model did not significantly change the observed results.

## Discussion

This is the first study to demonstrate six-year effects of a mindfulness-based intervention. Results indicate that medical and psychology students receiving mindfulness instruction in their first or second year of training reported increased well-being at six-year follow-up. Furthermore, the intervention group reported greater increases in the trajectory of DM and PFC, along with greater decreases in the trajectory of AFC over the six-year period. The effect on PFC yielded substantive increases in well-being. The effects were found despite poor to moderate adherence to formal mindfulness practice.

The short-term strengthening of DM [1–3], and well-being [52, 53], have been well documented in mindfulness intervention research. For instance, in non-clinical samples (i.e. physicians, university students, and healthy adults), studies with a 12-month follow-up period have documented sustained effects on both DM and positive psychological outcomes (i.e. relaxation, subjective well-being, self-compassion, and empathy) [54, 55]. The current study extends extant findings, offering novel evidence demonstrating effects in well-being six years after a mindfulness intervention. Intervention effects are striking, given the relatively low dose of the intervention (a 15-hour course over seven weeks plus 8 x 1 ½ hour booster sessions over four years), the fact that almost half of the students declined to participate in booster sessions offered once a term, and, the intervention group’s decline in frequency of home mindfulness practice over time in contrast with the control group’s increase in practice. While the number of students that reported practicing mindfulness was higher in the intervention group at all time points, better adherence to practice in the intervention group might have led to more robust results, as frequency of formal mindfulness practice has been shown to predict levels of mental distress in the short term [41], and dispositional mindfulness in the long term [40]. Another factor that could have influenced the results was the higher baseline levels of AFC in the dropouts compared to the completers. Those students high in avoidance could have benefited more from the intervention, but it is also possible that they would not have shown increases in PFC. Attending to avoidance as a predictor of attrition in future studies of MBI’s may be worthwhile. In line with previous findings [56, 57], one possible explanation for the mindfulness intervention’s long-term effect may have been that it facilitated a shift in the way students coped with adversity. An overarching mechanism of mindfulness training is improved self-regulation through more developed attentional control, emotion regulation and self-awareness [58]. The current study chose to examine the influence of mindfulness training on emotion regulation by exploring coping strategies theorized to be differentially associated with both well-being and mindfulness, namely AFC, PFC and SS. However, SS was non-significant in the multivariate LGC model. This may have been due to the fact that this scale contained both problem-focused and avoidance focused coping questions. The AFC and PFC strategies are antithetical and trigger distinct down-stream cognitive, emotional, and behavioral consequences. Results from this study support this claim, suggesting that, as problem-focused strategies become more common over time, reliance on avoidance-focused strategies declines. These findings, in concert with previously reported results from the same study sample, suggest that mindfulness training has both short term [39, 41], and long-term effects on coping, reducing reports of AFC and increasing reports of PFC. This is in accordance with a RCT with 202 adults that demonstrated that a brief mindful acceptance induction produced more approach and less avoidance coping than relaxation and self-affirmation controls [59].

The relationship between mindfulness, cognitive forms of approach-focused coping (i.e., positive reappraisal), and well-being has been rigorously characterized in the Mindfulness to Meaning Theory (MMT) [60]–which asserts that mindfulness training augments cognitive coping to boost well-being by virtue of its effects on deautomatizing maladaptive schemas and enhancing psychological flexibility. Findings from this study support the MMT’s core, longitudinal claims, in that mindfulness training was shown to significantly improve PFC, which in turn impacts well-being six years after the intervention. Additionally, increases in DM were associated with increases in PFC and increases in both DM and PFC were associated with decreases in AFC over the observed six-year time period. As such, these processes may be mutually intertwined and positively influence one another–a hypothesis outlined in the MMT.

Furthermore, despite low levels of formal mindfulness training and practice, longitudinal intervention effects raise questions as to the optimal focus of mindfulness interventions–a consideration also raised in two recent meta-analyses [52, 61]. The current study’s intervention included teachings and experiential exercises exploring the value of self-acceptance, tolerance of thought and feelings, and notions such as “I am not my thoughts”, which is in keeping with mechanisms of third generation cognitive therapies such as Acceptance and Commitment Therapy. These concepts and teachings may not rely on repeated formal mindfulness practice to remain valuable, a notion that is supported by the fact that amount of formal practice did not predict 6-year outcomes. A salient research question is therefore whether MBSR would be as effective with less emphasis on formal mindfulness practice, and conversely, whether other interventions would improve with the inclusion of mindfulness practice. Furthermore, intention and attitude in practicing mindfulness could be as important as the amount of practice completed [62], and motivational factors and the quality of mindfulness practice should be attended to in future research.

Increases in the control group’s frequency of formal mindfulness practice and in their DM over time, could indicate that self-selection may have influenced this study’s results. All participants had agreed to participate in this study hoping to receive mindfulness training, and many pursued mindfulness training on their own during the follow-up period. Hence, the presence of “contamination” as a cause of weakening the effects observed in mental distress in the four-year follow-up cannot be ruled out [40]. This may also have weakened the strength of the current findings. Furthermore, participants were young, predominantly white medical and psychology students. As such, our results may not be valid for other age or socio-cultural groups, or for people less educated and motivated to take part in a mindfulness course.

Other limitations to this study should be noted. The lack of an active control condition made it impossible to control for non-specific factors, such as support from the group or leaders, or different placebo effects. However, the longitudinal intervention effect in mindfulness disposition is an indication of intervention-specific active ingredients. Another limitation is the sole use of self-report instruments, which may introduce response bias such as social desirability, impression management, and memory bias. Future research could combine self-reporting and qualitative methods with physiological measurements (including brain imaging), objective cognitive measures, behavioural experiments, or judgments from significant others.

The strengths of our study include the rigorous RCT design. All reporting was done online and anonymously, without the experimenters knowing the identity of protocol codes. The study has a longer follow-up period than any other mindfulness study in the literature. Despite the need for replication studies and more sophisticated methods to disentangle effects and causal mechanisms [63], our findings support the value of using an abridged MBSR intervention to promote medical and psychology students’ health and resilience to cope with the stress of student life and the expected future professional challenges they may face. Integrating mindfulness training into higher education holds promise as a way of fostering the personal qualities future health care workers need to thrive and cope well with stressors. The small investment in time that such a course entails, in relation to the comprehensive six-year study curricula, begs the question why we do not offer this to health profession students in the same way that universities allocate resources for students to maintain and strengthen their physical health.

## Supporting information

S1 Protocol Study protocol. (DOC)

S1 Text Principal component analysis of WCCL. (DOCX)

S1 Table CONSORT checklist. (DOC)

S1 Dataset Mindful_coping_data. (SAV)
