# Supplementary material for: Six-year positive effects of a mindfulness-based intervention on mindfulness, coping and well-being in medical and psychology students; Results from a randomized controlled trial
Source: PLoS One. 2018 Apr 24;13(4):e0196053. doi: 10.1371/journal.pone.0196053 (PMC5916495; doi:10.1371/journal.pone.0196053)
Supplement: S1 Protocol — (DOC) [file pone.0196053.s001.doc]

# Mindfulness training for stress management: A two-centre randomised controlled study of medical and psychology students with long-term follow up

**Summary**

This study will assess the short and long term effects of a group-based mindfulness programme (Mindfulness-Based Stress Reduction, MBSR) on first year medical and psychology students at the Universities of Oslo and Tromsø.

Several studies have documented high levels of mental distress and low life satisfaction in students (1-3), and health care professionals (4,5), and the need to address this through teaching relevant coping skills (6,7). Optimal patient care is dependant on health care workers who are healthy, and able to cope with the strains of the helping professions. They must also have developed the ability to be fully present with their patients in order to see, understand and communicate well with them.

The primary outcome variables are mental distress, student stress and subsequent work stress, subjective wellbeing, empathy and mindfulness. We will also study explanatory moderator and mediator variables.

The study will be a two-centre randomized controlled study involving approximately 190 medical and psychology students from the University of Oslo and 135 from the University of Tromsø. The sample size calculation is based on a reduction in mental distress and perceived medical/psychology school stress of 20% in the intervention group.The control group will not receive an intervention. After the initial seven week course the intervention group will receive a follow-up session of 1,5 hours twice a year throughout their study course of 5-6 years. The follow-up period will last for 6 years in the Oslo cohort and last until one year after graduation. In the Tromsø cohort the follow-up period will be for 8 years and last until 3 years after graduation.

**Introduction**

### Mental distress and quality of life

The Health Service for students in Oslo report that an increasing number of students need professional help due to mental distress (8,9), and a National survey from 2005 shows that 13% of students in Norway have impaired mental health, compared to 8 % in the general population (10). In a study of Norwegian medical students one third reported mental health problems during the first 3 undergraduate years (11). Other studies have shown that psychology students also have high levels of emotional disturbances (3, 12). In one study on 287 American clinical psychology trainees the estimated prevalence of psychological distress, as measured by the General Health Questionnaire, was 59% (3). Both individual factors, such as previous problems, personality, negative life events, social factors, contextual stress and coping styles contribute (3, 5,11). Studies also show a strong association between emotional problems and life satisfaction (13).

A longitudinal study of Norwegian doctors found an increase in mental health problems in need of treatment over the years, from 11% during the first year after graduation to 17% in the fourth year (14). After 10 years follow-up 22% reported a need for treatment, indicating a persistent increase in prevalence (15).

Burn-out, a marker of mental distress, has been shown to be prevalent in medical doctors (16), and psychologists (17), but studies also show that it occurs in medical students with increasing prevalence during their studies (18).

### Study and Work-related stress

Several studies indicate that reported student stress and work stress have a negative influence on the health of students and health care workers. It is associated with reduced life satisfaction (6), increased rates of depression (19-21), reduced job-satisfaction (22), disrupted personal relationships (23), suicidal behaviour (24, 25) and burn-out (26). This in turn has a negative effect on their academic performance (27) and on the quality of care their patients receive (28).

### Coping with stress, Self-regulation and Metacognition

Over the last 20-30 years there has been an increasing focus on the way we cope with our life challenges, and studies show that this is important for the quality of life (6) and mental health for students and health professionals (27,29). According to self-regulation theories developed during the last 20 years, individuals regulate behavior in ways aimed at attaining desired goals (promotion) or avoiding undesired goals or outcome (prevention) (30). This is relevant both regarding academic motivation (31) and emotional distress (32). Another concept relevant to coping is metacognition. This refers to the psychological structures and processes involved in control, modification and interpretation of thinking itself, and these seem to play a central role in information processing and coping (33).

### Personal development and mindfulness

The personal quality of doctors and psychologists is of importance to all patient encounters and has substantial effect on the outcome of psychotherapy (34). In spite of this, the personal development aspect is given limited emphasis in the curriculum of medicine and psychology. Studies show that empathy in medical students decrease during their studies (35). Similar studies with psychology students are not identified, but one study comparing empathy in psychology interns and medical residents showed higher levels of empathy among psychologists (36). The ability to be empathic is a key factor in good patient care. Personal distress and decreased empathy are associated with increased odds of future self-perceived errors (37).

Other personal characteristics of good helpers are their ability to be fully present and acknowledging to the patient, tolerating the emotions that arise in themselves and the people they care for, feeling empathy and compassion, being open to experience, and being able to accept ones` own thoughts and experiences as a helper. These are all personal qualities that are closely related to mindfulness (38-40).

Training in mindfulness has over the last 20 years been used as a method for personal development and coping with mental processes. A recent textbook summarizes the evidence base and applications of these approaches within psychotherapy (41).

**Previous research on MBSR**

### Mental distress and quality of life

A non-randomized study of MBSR involving 300 second year American medical students, where the control group received a course in complementary medicine, showed significant reduction in mood disturbance, stress, anxiety, confusion and fatigue in the intervention group after the intervention (42). A recent review of research on medical student distress emphasised the need for research concerning factors that promote well-being (43). Several factors influence quality of life in student populations. Some studies have shown that personality plays a modest part, while social support and coping styles are more important (5, 6).

**Study and work-related stress**

A randomized MBSR study involving 78 pre-clinical and clinical medical students showed a significant reduction in psychological stress measured at exam time in addition to reduced anxiety and depression (44). Reduction in job stress was found in another randomized MBSR study for 38 health professionals including doctors and psychologists; in addition to increased self-compassion, and less psychological symptoms of burn-out (45).

In a study of medical students (6) it was found that a low level of passive, emotion focused coping style (wishful thinking) was associated with stable high life satisfaction. Studies of mindfulness training have shown that it leads to changes in coping style (46-48), and it is hypothesized that this occurs as a process of self-regulation (49).

### Personal development and MBSR

The MBSR study involving 78 pre-clinical and clinical medical students also showed significant increase in empathy while empathy decreased in the control group (44). Another recent study found significant improvements in mindfulness and spirituality among participants in a MBSR program and these changes were associated with reduction in psychological distress (41).

**Aims of the study**

The aim of the investigation is to study the proximal and distal impact of an MBSR intervention on outcome parameters measuring mental signs of stress and burnout, subjective wellbeing, empathy and mindfulness among medical and psychology students.

The following factors will be investigated as explanatory variables:

- Demographics (age, gender, civil status)
- Personal information on personality, coping style, self-regulation, perceived social support and life events
- Attendance at the MBSR classes
- Practice of mindfulness exercises

**Qualitative measures**

Studying processes and mechanisms of mindfulness using standardized measures of outcome and explanatory factors may not capture the full range of experiences. Thus, to compensate for this possible limitation, a qualitative approach will be included to explore immediate and long term experiences related to attending the MBSR courses by performing group interviews with medical and psychology students 3 months and 2 years after the intervention.

**The contribution from this study**

The study design will allow us to compare the effects of the intervention on medical and psychology students and analyze differences relating to the outcomes. We will further investigate to what extent the results are achieved in different student populations with different intervention instructors, and thus be able to assess the extent to which the study results can be generalized.

We will also investigate to what extent the MBSR intervention has a long term effect on mental distress and subjective wellbeing and how an intervention during the first year of studies influences future student and job stress and symptoms of burn-out. This has not been done before.

This study will investigate in what way the coping factors of self-regulation and coping style influence the effect measures, if they change as a result of the intervention, and how these changes are correlated with the other outcome measures. This has not been done before. It will also allow us to better understand the mechanism of the mindfulness intervention effects.

We will also examine personal development by measuring whether the intervention does lead to increased empathy and mindfulness. This is the first study to monitor how a MBSR intervention affects personal development over a long follow-up period.

**Hypotheses to be tested**

1. MBSR decreases mental distress.
2. MBSR decreases burnout.
3. MBSR increases subjective wellbeing.
4. MBSR decreases student stress.
5. MBSR decreases future job stress.
6. MBSR increases empathy.
7. MBSR increases mindfulness.
8. MBSR leads to less passive coping and more active coping skills.
9. MBSR increases promotion-focused and decreases prevention-focused self-regulation.
10. The effect of MBSR is positively related to degree of reported mindfulness training.
11. The effect of MBSR is positively related to the degree of reported stress and mental distress.
12. The effect of MBSR is negatively influenced by life events.
13. The effect of MBSR is positively influenced by degree of social support.
14. The effect of MBSR is independent of gender.
15. The effect of MBSR is positively influenced by being married or cohabitating.
16. The effect of MBSR is positively related to neuroticism and conscientiousness and negatively related to extroversion at the start of the studies.
17. MBSR reduces neuroticism and conscientiousness measured at the end of the studies.
18. The effect of MBSR is positively influenced by initial levels of prevention-focused self-regulation in those who complete the training program.
19. Initial prevention-focused self-regulation is associated with drop-outs and less adherence with the MBSR training principles.
20. Increased promotion-focused self-regulation reduces negative affect.

The statistical procedures to test these hypotheses are described below (see paragraph “Analysis strategies”).

**Overview of the trial**

The study has four elements:

1) We will first carry out a systematic review (registered with The Campbell Collaboration) to create an overview of the current evidence on the effects of MBSR on all study populations judged from randomized controlled trials. The aim is to establish the evidence base for this intervention, and also get input on factors we need to pay attention to when giving the MBSR intervention. It will also allow us to make hypotheses that will guide the statistical analyses of the results of the trial. A paper will also be written elaborating and discussing the theoretical framework for mindfulness-based interventions.

2) We will undertake group interviews with medical and psychology students in Oslo from different stages of their studies. The aim is to get their feed-back on how best to adjust the intervention to their studies. This will be undertaken from March to June 2009.

3) The main study will be done as a randomized controlled trial. In Oslo this will take place in the autumn of 2009 and spring of 2010 with 190 students. The University of Tromsø in the North of Norway will undertake a similar study in 2010 and 2011, including 135 students of medicine and psychology, using the same design and protocol but with independent project organization, MBSR instructors and financing. The data, obtained using validated questionnaires, from both arms of the trial will be used for comparative analysis.

4) We will undertake group interviews with a group of medical and a group of psychology students from the intervention group in Oslo 3 months and 2 years after the intervention (T3) in order to get a qualitative feedback on their experiences with the MBSR intervention. This can also contribute to the understanding of the active mechanisms of effect and generate hypotheses for future studies. The qualitative parts of the study are by nature explorative, and thus it is not relevant to set up specific predictions.

The control group will continue their studies without any special intervention. The MBSR is a new intervention into an existing curriculum. Establishing an active intervention for the control group will make it more difficult to assess whether the outcomes in the MBSR intervention group is different from the normal curriculum control group.

There is a danger for contamination and interested students in the control group might want to learn the same mindfulness skills as the intervention group is taught. This is difficult to prevent, but by doing sub-group analysis looking at to what extent students in both groups practice mindfulness exercises, and measuring their level and development of mindfulness, we will be able to evaluate the relationship between mindfulness training, mindfulness and the other outcome measures.

**Trial population**

*Trial site(s) and population(s)*

All 2^nd^ term medical and 2^nd^ term professional psychology students autumn 2009 and spring 2010 at the University of Oslo will be our study populations. The Tromsø study sample will be recruited from all 2^nd^ term medical and 2^nd^ term professional psychology students in spring of 2010 and 2011. The reason for choosing 2nd term psychology students is that many students are exempt from the first term because of previous courses. The second term is the first period the whole class is together. These classes consist of a total of 400 medical students and 160 psychology students and they will be invited to participate. We hope to include approximately 105 psychology students and 220 medical students in the study.

*Inclusion and exclusion criteria*

The whole study population will be invited to participate. There will be no exclusion criteria.

*Sources or methods of recruitment*

The project manager will meet with the students` classes to inform them of the study and invite them to participate. In Tromsø the project manager Ida Solhaug will co-operate with the students’ health service at the University in providing information about the study and recruiting participants. The participants will have the chance to ask about any queries they have before deciding to participate. Inclusion will be done separately for each class of medical and psychology students, totally six times.

*Information for participants*

Information about the study will be given in the student classes by the project managers in Oslo and Tromsø respectively. In addition all students will receive a letter of invitation explaining the study with a deadline date for entering the study and containing a link to a web-site where they can give their online consent within a preset date.

**Allocation of interventions**

*Methods for randomization*

After inclusion and filling out the baseline protocol, the database will perform randomization using a computerized program. It will be done separately for each class to secure equal numbers of medical and psychology students from each class entering the intervention and control group.

*Methods for concealment of allocation*

Randomization will be done by the computer database at the Norwegian Knowledge Centre for the Health Services, based on the included participants from each class. Each student will receive a number-code to be used on all protocols and the code key linking the student number code and name will be kept a technical staff at the Knowledge Centre not involved in the study. A reply will be sent automatically from the database program, after baseline protocols are filled in, to each student by e-mail confirming whether he/she is in the intervention or control group. The student ID code will be used by the student when entering the database to fill in the study protocols, and by the project manager when communicating with the students.

**The interventions**

*Description of intervention and intervention delivery*

MBSR is a 7 week programme in mindfulness training that will be given to groups of 20-30 students. It has 6 weekly sessions of 1,5 hours and one 6 hour session after seven weeks. The main elements in the program are: 1) Simple physical and mental exercises to increase mindfulness of what one experiences in the present moment. 2) Short teachings on mindfulness, stress, stress management and mindful communication supported by a course manual and CDs with mindfulness exercises for home practice. 3) A group process reflecting on ones` experiences while practicing mindfulness at home and in the classes. About one half of the time in the classes is devoted to this. Emphasis is on creating an open, curious, nonjudgmental and accepting attitude to all experiences. The weekly sessions has a standardized programme with the following main contents (50):

***Week 1*:** Mindfulness and the power of being present.

*Exercises:* Sensing exercises like eating a raisin slowly and mindfully, and simple physical anchoring exercises. Body scan, lying down, focusing the attention sequentially on different parts of the body from the toes upwards to the head, noticing the sensations that are present with openness and curiosity, but without trying to change them. Body scan is practiced during sessions 1,2 and 7. *Teaching:* What is mindfulness? *Homework:* Body scan 6 days a week, and practicing doing a daily activity mindfully, like brushing the teeth or taking a shower*.*

***Week 2:*** Perception – how we perceive reality.

*Exercises:* Sitting meditation directing the attention to the movement of breathing. When the mind wanders, one accepts this and then gently returns the attention to the breath.

This is practiced during sessions 2-7, for periods from 10-30 minutes and is assigned for homework most weeks. *Teaching:* Limitations to perception, its influence on our understanding and actions, and the effect of mindfulness on our perception of realty. *Homework:* Body scan 6 days a week. 5-10 minutes sitting meditaion. Practice doing a daily activity mindfully. Noticing a pleasant event each day and paying attention to sensations, thoughts and feelings that arise, and taking a note of this.

***Week 3:*** Stress and how it affects us.

*Exercises:* Simple physical exercises from the yoga tradition done mindfully, paying attention to the sensations in the body and the breathing, without straining or forcing oneself. Walking meditation; walking slowly while focusing on sensations in the body, and gently bringing the attention back when it wanders.. *Teaching:* Stress and its effects on the mind and the body. *Homework:* Alternate Yoga and sitting meditation 6 days a week. Noticing unpleasant and stressful situations and paying attention to sensations, thoughts and feelings that arise without trying to change anything.

***Week 4:*** Coping with stress

*Exercises:* Yoga, sitting and walking meditation. *Teaching:* Stress response versus reaction, using mindfulness as a tool. *Homework:* Alternate sitting meditation with Yoga or body scan 6 days a week. Noticing stressful situations and using mindfulness to explore new ways of responding.

***Week 5:*** Communication

*Exercises:* Yoga, sitting and walking meditation. Communication exercise. *Teaching:* Mindful communication. . *Homework:* Alternate sitting meditation with Yoga or body scan 6 days a week. Using mindfulness to explore new ways of communicating.

***Week 6:*** Self-reliance

*Exercises:* Sitting and walking meditation. *Teaching:* On acceptance and trust in one-self and life. *Homework:* Alternate mindfulness exercise with and without CD instruction 6 days a week. Exploring an open and trusting attitude to life. Examining one’s daily habits concerning life-style.

***Week 7:*** ***6 hour mindfulness session***.

A day program of different mindfulness exercises spent in silence except for instructions. The last 45 minutes for group reflection and evaluation of the program.

The programme has been translated into Norwegian (51) by the principal investigator, who has tried it out in a randomized control trial in family practice with good results for patients suffering from stress and chronic illnesses (52).

All courses in Oslo will be led by the principal investigator in Oslo who is a medical doctor and has practiced mindfulness for 30 years. He spent three months as an intern at the MBSR centre in Massachusetts in 2002. His assistant will be a young psychologist, Rebekka Egeland, who has practiced mindfulness for three years and who has writing a book in Norwegian on mindfulness. In Tromsø all courses will be led by the principal investigator in Tromsø, who is a psychologist well trained in implementing MBSR and a colleague who has received the same training.

The participants will receive a course manual and a double CD with mindfulness exercises for home practice. They will be encouraged to practice at home 30 minutes daily, but the main emphasis is on informal practice of mindfulness during daily life.

Since there will be approximately 45 students that will get the intervention each term in Oslo, and 65 in Tromsø, two or three MBSR classes will be organized each week. Whether we will mix medical and psychology students will be decided after the group interviews with the students. The students will sign up for the day of the week they favour and be encouraged to stick to this class. If they miss a class, they will be able to attend this class with another group to fill in their attendance in the programme. The intervention will be given in the afternoon outside normal lecture times.

After the course, the intervention groups will get a 1,5 hour follow up session twice a year. The students will be notified of the follow-up sessions well in advance by email. In Massachusetts, where the programme was developed, former participants are invited to attend all day sessions that are held 4 times a year to help them continue their practice. Continued reinforcement of mindfulness practice has been found to be important for the effects seen (53).

**Outcome assessment**

*Outcome measures*

**Mental distress** will be measured by three inventories. The General Health Questionnaire (GHQ-12) has been used widely internationally to measure mental distress, also in Norwegian student populations (54,55) The short form of Hopkins Symptom Checklist, the SCL-5 which examines anxiety and depression and has shown good specificity (82%) and sensitivity (96%) for detecting mental distress (56,57). It has been used in a Norwegian MBSR intervention study (52), and in an intervention study using coaching for Norwegian doctors with burnout (58). Burnout as an expression of mental distress will be measured by the Maslach Burnout Inventory. We will use the 16 item student version (MBI-SS) which measures exhaustion (5 items), cynicism (5 items) and efficacy (6 items). It has good psychometric properties and is tested in many cultures (59). After the students have qualified, we will use the normal MBI for health professionals that were used in a recent study of Norwegian doctors (58).

**Subjective well-being (SWB)** will be measured by using a short version of the SWB-scale developed by Moum et al in 1990 (60) and used in several studies (61, 62). It has shown good psychometric properties. The index is constructed using the sum-score of four items: a) “When you think about your life at present, would you say you are mostly satisfied with your life, or mostly dissatisfied? Seven response categories from extremely satisfied to extremely dissatisfied. This question has been used in other studies of Norwegian medical students, doctors, and in population surveys (5, 6, 63). b) “Are you usually happy or dejected?” The five categories range from happy to dejected; c) “Do you mostly feel strong and fit or tired and worn out?” The seven categories are from extremely strong and fit to extremely tired and worn out; d) “Over the last 14 days have you suffered from nervousness or restlessness?” The four categories range from not at all to very much. The sum of each question is divided by the number of categories, and then the four questions are summed up to one score. This index correlates highly with validated quality of life measures (61), and its construction conforms to the generally accepted operationalisation of subjective well-being (61, 62) comprising a cognitive aspect (life satisfaction), as well as positive affect (happy and strong) and negative affect (nervous and tired).

**Stress:**

**Student stress** will be measured by the 13-item Perceived Medical School Stress (PMSS) developed by Vitaliano (64) and used in studies of stress in student populations (6, 65). It will be slightly adapted to fit both student populations.

**Job stress** will be measured after qualifying by using a modified version of Cooper`s Job Stress Questionnaire (CJSQ) which has been used in a nationwide Norwegian prospective cohort study on the impact of job stress and working conditions on mental health problems among young doctors (20), and also in an intervention study among Norwegian doctors (58).

**Personal development**

**Empathy** will be measured by the 20-item Jefferson Empathy Scale (JSPE-HP version). The 20 questions are answered on a seven-point scale from strongly disagree to strongly agree. It was developed for medical students and health professionals and has good psychometric properties (66, 67). The health professional version measures empathic behavior as 3 factors: a) the ability to take the patient’s perspective, b) the ability to give compassionate care and c) the ability to stand in the patient’s shoes. It will be translated into Norwegian using a standardized two way procedure, and pilot-tested in a student population.

**Mindfulness** will be measured by using the 39-item Five Facet Mindfulness Scale (FFMS) which measures five facets of mindfulness and has good psychometric properties (37, 68). The five facets are a) the ability to observe, b) to describe and c) to act with awareness, and d) the ability to be fully present with an attitude of non-judgment and e) non-reactivity towards what arises in oneself and in the situation one is in. The FFMS is shown in student populations to be strongly positively correlated with meditation experience, openness to experience, emotional intelligence and self-compassion (p<0.001), and strongly negatively correlated with psychological symptoms, neuroticism, thought suppression, difficulties in emotional regulation, alexitymi, dissociation, experiential avoidance and absent-mindedness (p<0.001) (37).

*Mediating and moderating factors*

**Personality** will be measured by the 27-item Basic Character Inventory (BCI) (69, 70). It measures the personality dimensions of neuroticism, extraversion and conscientiousness, which has been shown to be related to stress in health professionals (5, 71).

**Perceived social support** will be measured by five questions used in other Norwegian studies involving medical students and doctors and which has been shown to influence subjective well-being (5). The five questions measure: a) frequency of contact with close friends, b) appreciation from friends outside family, c) presence of warm and caring confidents, d) degree of affiliation for groups, and e) anticipated support if they should fall ill. All items are scored using five response categories, with higher scores representing higher levels of experienced support. All items sum up to one variable with a possible range from 5-25, and studies have shown acceptable Chronbach alpha of 0.70 (5).

**Life events**

Negative life events in the last year have been shown to be negatively correlated to mental health and life satisfaction (5, 11, 20) and we will include three questions regarding a) serious disease/accident/hospital admission, divorce/separation/broken relationship and c) serious illness/death of family member or close friend. In addition positive life events has also been shown to be related to burnout in medical students (18), and two other items of importance will be included from the demographic variables; namely d) getting married and e) having a child. Each question has a score of having (1) or not having (0) experienced this in the last year. The items sum up to one variable with a possible range from 0-5.

**Coping** is measured by the 42-item Ways of Coping Checklist (WCCL) (72) which has good psychometric properties and has been used in Norwegian student populations (6). It was originally divided into five coping dimensions (problem focused, self-blaming, seeking social support, wishful thinking and avoidance). A previous study on Norwegian medical students showed a three-factor solution of problem focused, seeking social support and wishful thinking modes of coping (6). Such analysis will also be done in our study to identify the relevant coping strategies. We will analyze the relationship between coping style at inclusion and outcomes, how coping styles change during the study and how this change relates to the outcomes, and whether the changes in coping style persist over time.

**Regulatory focus (RF)** will be measured by the 18-item Regulatory Focus Questionnaire consisting of two subscales measuring promotion and prevention goals (30). Psychometric properties have been good (73). A promotion focus is associated with openness towards ones own life-goals and change, a high tolerance for failure, and a tendency to take risks. A prevention focus is associated with skepticism to change, preference for stability, a low tolerance for failure, and a tendency to avoid risks. It has been suggested that depression is a result of a failure in the promotion regulatory system (32). Self-regulation has also been associated with academic motivation: Promotion-focused individuals are more inspired by positive role models who highlight strategies for achieving success whereas prevention-focused individuals have been most motivated by negative role models highlighting strategies for avoiding failure (31). We will analyze the relationship between initial levels of RF and the outcomes and drop-out tendency, how RF change during the study and how this change relates to the outcomes.

**Age, gender, marital status and number of children**

Marital status will be coded as single or living with partner/wife.

**Reported mindfulness practice** will be measured by four questions used in other MBSR studies (51). These are a) “How often do you practice mindfulness exercises (body-scan, relaxation, yoga, Tai Chi, Qi Gong or meditation)?” The categories are: daily, 4-6 times a week, 1-3 times a week, once per 14 days, 1 per month, never. b) “When you practice, how much time do you normally spend?” The categories are 0-10 minutes, 11-20 minutes, 21-30 minutes, 30-45 minutes, 45-60 minutes. c) “How often do you practice mindfulness of breathing during everyday life?” The categories are from 6-1: daily, 4-6 times a week, 1-3 times a week, once per 14 days, 1 per month, never. d) “How often do you practice mindfulness of daily activities (eating, walking, showering etc)?” The categories are from 6-1: daily, 4-6 times a week, 1-3 times a week, once per 14 days, 1 per month, never. The two first questions will be transformed into minutes by multiplying frequency by number of minutes. The two last questions, will be summed up and divided by two to give an estimate of informal mindfulness practice ranging from 2-12.

Some studies have found a correlation between reported mindfulness training and the effects measured (51, 74), while other studies have not shown this. We will therefore assess the extent to which the intervention group practices mindfulness exercises and see how this relates to the outcome measures.

*Timing of outcome assessment*

190 students randomized 2009 and 2010

T1-protocol before the intervention

95 intervention group

95 control group

T2-protocol after the intervention

T3-protocol 2 years after the intervention

T4-protocol before finals

95 gets one 3 hour session twice a year throughout studies

T5-protocol 1 year after finished study, 2016-17

95 continue study as normal

95 doctors and psychologists

95 doctors and psychologists

**Flow-chart of the study in Oslo**

At T1 all measures are included.

At T2 all measures are included except burnout, age, sex and personality (BCI).

At T3 all measures are included except burnout, age and sex.

At T4 all measures are included except age and sex and personality (BCI).

At T5 all measures are included except age and sex. In addition job stress (CJSQ) will be measured in stead of student stress (PMSS).

135 students randomized 2010 and 2011

T1-protocol before the intervention

68 intervention group

67 control group

T2-protocol after the intervention

T3-protocol 2 years after the intervention, T4-protocol before final exam

68 gets one 1,5 hour session twice a year throughout studies

T5-protocol 1 year after finished study, 2017-18

67 continue study as normal

68 doctors and psychologists

67 doctors and psychologists

T6-protocol 3 years after finished study, 2019-20

**Flow-chart of the study in Tromsø**

At T1 all measures are included.

At T2 all measures are included except burnout, age, sex and personality (BCI).

At T3 all measures are included except burnout, age and sex.

At T4 all measures are included except age and sex and personality (BCI).

At T5 all measures are included except age and sex. In addition job stress (CJSQ) will be measured in stead of student stress (PMSS).

At T6 all measures are included except age, sex and personality (BCI). Job stress (CJSQ) will be measured in stead of student stress (PMSS).

**Post-recruitment retention strategies**

*Participant retention:*

All the students entering the study will receive payment for filling out the protocols. The time they spend on this will be 45 minutes each time, and since half the participants will not receive any intervention, compensating them for the time spent seems fair and will hopefully lead to less attrition. The control group will receive 350 kroner per protocol and the intervention group 250 kroner. The first payment will be given after T1 and T2 protocols are filled in. We will also send the participants in the intervention and control group a news-sheet together with notice for filling in follow-up protocols at T3-6 in order to keep up the interest for the study. This will contain information about topics relevant to the study, but no study results. The database system will automatically send out three reminders to all participants for missing protocols at each collection point and reminders if protocols are insufficiently filled in. The control group participants will be offered a free course in MBSR when the study is completed.

We will monitor compliance by noting attendance at all group meetings, giving a score from 1-7 and by self-reporting of mindfulness practice by the participants. Any protocol deviations will be documented along with any adverse events.

Any contamination in the control group will be documented through answers to questions on what mindfulness practices they are doing. Losses and dropouts will be fully documented.

**Safety monitoring and adverse events**

*Data and safety monitoring*

There has not been reported any adverse events in MBSR trials with student populations, and this is unlikely to occur. As the principal investigators are leading all intervention groups it will be easy to pick up any unforeseen events and act on these. Severe mental illness (psychotic illnesses) represents a relative contradiction to MBSR, but the students entering the trial are unlikely to suffer from this. Hence there will not be established interim analysis plans or statistical stopping rules for safety.

**Data collection and management**

*Data collection*

Data will be collected by questionnaires given at T1-6 to both intervention and control group. In addition a group interview with medical and psychology students will be undertaken before T1 with a mixed group of students from different student classes that will not enter the study and after T3 with groups of students that have received the intervention.

The following data will be collected:

|  | T1 pre-intervention | T2 post-intervention | T3 2 yr post- intervention | T4- Before final exam | T5- 1 yr after final exam | T6-3  yr  After  Final  exam |
| --- | --- | --- | --- | --- | --- | --- |
| Focus group |  |  | X |  |  |  |
| Mental health GHQ-12 | X | X | X | X | X | X |
| Anxiety and depression (SLC-5) | X | X | X | X | X | X |
| Burnout  Maslach BI | X |  |  | X | X | X |
| Subjective wellbeing | X | X | X | X | x | X |
| Student stress PMSS | X | X | X | X |  |  |
| Work-stress CJSQ |  |  |  |  | x | X |
| Empathy JSPE | X | X | X | X | X | X |
| Mindfulness FFMS | X | X | X | X | X |  |
| Promotion/Prevention scale | X | X | X | X | X | X |
| Personality BCI | X | X | X | X | X |  |
| Perceived social support | X | X | X | X | X | X |
| Life events | X | X | X | X | X | X |
| Coping | X | X | X | X | X | X |
| Age and sex | X |  |  |  |  |  |
| Marital status | X | X | X | X | X | X |
| Number of children | X | X | X | X | X | X |

Consent will be secured from the Regional Ethical Committee and the Norwegian Data Inspectorate.

*Data management system*

The protocols will be filled in on internet. A database with the protocols will be created by the Informatics Department at the Norwegian Knowledge Centre for the Health Services. The students will log on using an ID code given to them at inclusion. The system will automatically send out reminders to the students about missing protocols or insufficiently filled in protocols. All questionnaire data will be stored on a computer without possibility of unjust external access. Materials from the qualitative analyses will be stored on audiotapes. Their content will be deleted after transcription, and the transcriptions will be made anonymous.

*Data entry*

The data will be automatically transferred from the protocols to excel spread-sheet and then by the research staff from excel to the statistical software program. After data entry the principal investigators will perform logic checks, range checks and coding checks on all entries.

*Quality control*

There will be single entry of data. Changes in entries will be logged and verified by a second researcher and queries communicated and resolved. The source of the change will be filed with the original data form.

*Progress reports*

A progress report will be sent to the funding body at the Norwegian Medical Association every six months on their specified form.

*Final reports*

A report will be written after the data from T3 has been analyzed, and we aim to publish the main findings at this time. The second face of the study will be carried out at T4 to T6 but a separate grant will be applied for to cover this part of the study.

**Sample size**

*Sample size justification*

The sample size calculation is based on an expected reduction in mental distress and perceived medical school stress of 20% in the intervention group. The sample size is calculated separately for each outcome measure, all using 80% power and 5% significance; two sided test.

**Mental distress** measured by GHQ-12 in 1^st^ year Norwegian university students is 10.63 (SD 5.08) (54) and in 3^rd^ year medical students 12.7 (SD 5.3) (55).

Assuming that the intervention will prevent an increase in mental distress measured by GHQ-12 from 1^st^ to 3^rd^ year, the sample size would have to be 104 in each group, but since the MBSR intervention is shown to improve mental distress in student populations (39), the necessary number is likely to be lower.

Mental distress measured by SCL-5 in a longitudinal study of 236 Norwegian medical students increased from a mean value of 2.6 (SD 3.0) in the first year to 4.2 (SD 4.4) in the last year. Assuming that the intervention can prevent this distress, the sample size in each group would have to be 85 (personal communication Reidar Tyssen).

**Stress:** For medical school stress measured by PMSS, a recent study (55) showed that 3^rd^ year Norwegian medical students had a value of 24.74 (SD 8.49) before and 19.74 (SD 8.04) 3 months after an intervention using self-development groups). Assuming the same effect for our intervention, the sample size would have to be 58 students in each group.

**Subjective wellbeing:**

Sample size is calculated on the basis of data from 210 medical students measured in their first year (1993) and last year (1999). The subjective wellbeing scale used included three of the four questions in our scale (not b – happy or dejected) and showed a reduction in wellbeing from 2.39 (SD .35) to 2.20 (SD .46) (personal communication Reidar Tyssen), giving a sample size of 70 in each group.

**Personal development:**

**Empathy** will be measured by JSPE, and in a study of American medical students (34) empathy fell from 123.1(SD 9.9) at the beginning to 120.9 (SD 13.9) at the end of the 3rd year. Other studies have shown that empathy starts falling in the first year of medical studies (45), and assuming that a total fall over the 3 first years of 6.0 (SD 10) could be prevented as a result of the MBSR intervention (studies have shown increase in empathy as a result of MBSR (45)), the sample size would have to be 45 in each group.

**Mindfulness** measured by FFMS measures five facets of mindfulness, and a recent study comparing mindfulness in a student population with a population of mindfulness practitioners (37) report differences in the five facets from 2.77 (SD 5.77) to 7.64 (SD 4.84). Assuming a similar difference in the intervention and control group gives sample sizes of 69 to 11 in each group.

We intend to include 95 students in the intervention group and 95 in the control group in Oslo, and 68 students in the intervention group and 67 in the control group in Tromsø. Even with improvements in the mental distress, QoL, stress and empathy in the control group (although this is not likely based on longitudinal studies on Norwegian medical students (6)), and attrition to 70 students in each group in Oslo, and 50 in Tromsø, the sample size should be sufficient to generate significant results.

**Analysis strategies**

*Statistical analysis*

The analysis of the primary outcomes will follow the intention to treat principle – the participants will remain in the group they were randomized to.

We will use mixed model (multilevel) repeated measures statistics to assess change in outcome measures over time. Regression analysis including multivariate models will be used to identify predictor variables. The analysis will be guided by our hypotheses. We will use beta values from regression analyses as a measure of effect. In analyzing change, we will adjust for baseline values in outcome measures.

To assess difference between the intervention and control group at baseline we will use Chi-Squared test for categorical data and t-tests for continuous data.

As we will analyze with mixed model (multilevel) repeated measures statistics, missing observations will be handled by the statistical analyses. We will collaborate with statistical expertise at the University of Oslo and at the Norwegian Knowledge Centre for the Health Services.

Data from the qualitative parts of the study will be analyzed using grounded theory content analysis as well as discursive and narrative analytic strategies, to develop an understanding of the mechanisms of effect grounded by the knowledge and experience of the participants.

**Ethical aspects**

*Participant consent*

In the Tromsø part of the study care is taken to secure an unbiased and independent running of the program as members of the steering committee also have a role as professors at the faculty from which students are recruited. Thus, it is important to avoid a mixing of roles and conflicts of interest. Moreover, to avoid this kind of problem, qualitative interviews run from the Tromsø part of the study will be performed using informants from the Oslo part of the study. In Oslo the principal investigator is not employed at the university or involved in teaching the students. The other members of the steering committee in Oslo are faculty members, but these will not be involved in the practical running of the study.

**Trial management**

*Registering the trial*

The trial will be registered in the appropriate database via the Norwegian Medical Association and in Clinicatrial.org.

*Trial management*

The trial will be directed by a Steering Committee including the project manager in Oslo Michael de Vibe, professor Arild Bjørndal, associate professor Reidar Tyssen and associate professor Toril Dammen at the Medical faculty at the University of Oslo, and in Tromsø project manager Ida Solhaug, professor Tore Sørlie and professor Jan H. Rosenvinge. They will meet regularly to assess the progress of the trial.

The research project will be associated with the Research group on Professions at The Department of Behavioural Sciences in Medicine, Institute of Basic Medical Sciences, University of Oslo, and with the Norwegian Knowledge Centre for the Health Services. In Tromsø, the study will be run through the Student Counselling Center at the University, in order to secure an unbiased and independent recruitment procedure.

The daily running of the trial will be done by the project managers in Oslo and Tromsø respectively. They will be responsible for:

i) recruitment of participants

ii) production of data collection forms and other appropriate documentation for the trial

iii) data collection and management

iv) giving the group intervention and the follow-up sessions to the intervention group

iv) data analyses

v) collection of adverse event data

vi) reporting to the funding organizations

We have convened a resource group for the Steering Committee, consisting of

Professor O Aasland, the Research Institute of the Norwegian Medical Association

PhD student Jon Vøllestad, faculty of Psychology, University of Bergen; Professor Ruth A Baer, University of Kentucky; Head of research J Carmody PhD, the Institute for Mindfulness in Medicine, Health Care and Society at the University of Massachusetts; research assistant Birigit Eliassen at the Mental Health Research Department at the University of Tromsø and clinical psychologist Antonia Sumbundu, Copenhagen.

They will advise on the trial as and when required.

*Research governance and good clinical practice*

- Ethics. The trial is approved by the Regional Ethical Committee and the Norwegian Data Inspectorate. Written consent by email will be obtained by all participants. All data forms will be coded to protect participant confidentiality. A focus group interview with students from both departments will be performed to involve participants in designing the research.
- Science. The design of the study follows the recommendations of the Trial Protocol Tool developed by the EC-funded Pragmatic Trials in Health Care (Practihc) project (www.practihc.org).
- Information. All research findings will be published and accessible
- Health & safety. Continual monitoring of possible adverse events due to the intervention will be undertaken
- Finance. Half yearly reports will be sent to the funding bodies for the research

**Economic evaluation**

The time resource used on the intervention will be assessed, and an evaluation of the cost benefit relating to the outcome of the study will be discussed in the publication of the results.

**Consumer involvement**

A group interview with medical and psychology students will be performed prior to the intervention in order to get their feed-back on how best to adjust the intervention to their studies, and to get their input on the design of the trial.

**Reporting, Dissemination and Notification of results**

*Publication policy*

The results will be published in peer reviewed journals. Suggested titles and authors:

1. Mindfulness Based Stress Reduction for improving Health, Quality of Life and Social Functioning in Adults: A systematic review of randomized controlled trials. de Vibe M, Kowalski K, Hammerstrøm KT, Bjørndal A.

2. Mindfulness in context: A theoretical overview of mindfulness-based interventions in late modernity. Solhaug, I., de Vibe M, Sørlie T, Rosenvinge J, Tyssen R, Dammen T, Bjørndal A.

3. Mindfulness training for stress management: A randomised controlled study of medical and psychology students. Study design and first outcome results. de Vibe M, Bjørndal A, Tyssen R, , Dammen T,Solhaug I, Rosenvinge J, Sørlie T.

4. Mindfulness training for stress management: A randomised controlled study of medical and psychology students. Two year follow-up results. de Vibe M, Solhaug I, Bjørndal A, Tyssen R., Dammen T, Sørlie T, Rosenvinge J.

5. Mindfulness training for stress management: A randomised controlled study of medical and psychology students. Comparative analysis of results in different study populations. de Vibe M, Solhaug I, Bjørndal A, Tyssen R,Dammen T, Rosenvinge J, Sørlie T.

6. Mindfulness training for stress management: The interplay between personality, self-regulation, metacogniton and outcome. Tyssen R, Dammen T, Sørlie T,Bjørndal A, de Vibe M, Solhaug I, Rosenvinge J.

7. The longitudinal effect on empathy and self-care after mindfulness training: A randomized controlled trial of students of medicine and psychology. de Vibe M, Solhaug I, Bjørndal A, Tyssen R, Dammen T, Rosenvinge J, Sørlie T.

8. Mindfulness training for stress management: A randomised controlled study of medical and psychology students. Short term outcome results (Tromsø cohort). Solhaug I, Sørlie T, Rosenvinge J, de Vibe M, Bjørndal A, Tyssen R.

9. Mindfulness training for stress management: A randomised controlled study of medical and psychology students. Three year follow-up results (Tromsø cohort). Solhaug I, Rosenvinge J, Sørlie T, de Vibe M, Bjørndal A, Tyssen R.

10. Mindfulness-Based Stress Reduction for medical and psychology students in two cohorts. A qualitative study. Solhaug I, de Vibe M, Rosenvinge J, Sørlie T, Tyssen R, Bjørndal A.

11. The relationship between self-regulation and negative affect: an intervention study. Rosenvinge J, Sørlie T, Solhaug I, Dammen T,de Vibe M, Bjørndal A, Tyssen R.

Article 1 will be published in 2009, article 2 will be published in 2010, article 3 in 2011, article 4-7 in 2012-16. In addition one or two articles will be written after completion of the trial.

*Disseminating the results*

The results will be presented at international conferences and spread in the Norwegian media.

**References**

1. Bramness JG, Fixdal TC, Vaglum P. Effect of medical school stress on the mental health of medical students in early and late clinical curriculum. Acta Psychiatr Scand 1991;84:340-5.
2. Dahlin M, Joneborg N, Runeson B. Stress and depression among medical students: a cross-sectional study. Med Educ 2005;39:594-604.
3. Cushway D. Stress in clinical psychology trainees. Br J Clin Psychol 1992;2:169-79.
4. Nylenna M, Gulbrandsen P, Førde R, et al. Unhappy doctors? A longitudinal study of life and job satisfaction among Norwegian doctors 1994-2002. BMC Health Serv Res 2005;5:44.
5. Tyssen R, Hem E, Gude T et al. Lower life satisfaction in physicians compared with a general population sample. A 10-year longitudinal, nationwide study of course and predictors. Soc Psychiatry Psychiatr Epidemiol 2009; 44:47-54
6. Kjellstadli K Tyssen R, Finset A et al. Life satisfaction and resilience in medical school – a six year longitudinal, nationwide and comparative study. BMC Medical Education 2006;6:48.
7. Shapiro SL, Schwartz DE, Schwartz GE. Stress management in medical education: a review of the literature. Acad Med 2000;75:748-59.
8. Olsen R, Øverli S. Helse og trivsel blant studenter ved Universitetet i Oslo 2003-2005. Rapport, Universitetet i Oslo 2008.
9. Sørensen K, Østvik AR, Lintvedt O, et al. Forebygging av psykisk helse blant studenter ved hjelp av et lavterskeltilbud på internet. Tidsskr Nor Psykologforen 2007; 44: 265-8.
10. Levekårsundersøkelsen. SBB 2005.
11. Midtgaard M, Ekeberg Ø, Vaglum P, et al. Mental health treatment for medical students: a national longitudinal study. Eur Psychiatry 2008;23:505-11.
12. Hudson SA, O'Regan J. Stress and the graduate psychology student. J Clin Psychol 1994;50:973-7.
13. Koivumaa-Honkanen H, Kaprio J, Honkanen R et al. Life satisfaction and depression in a 15-year follow-up of healthy adults. Soc Psychiatry Psychiatr Epidemiol 2004;39:994-9.
14. Tyssen R, Røvik JO, Vaglum P et al. Help-seeking for mental health problems among young physicians: is it the most ill that seeks help? A longitudinal and nationwide study. Soc Psychiatry Psychiatr Epidemiol 2004;39:989-93.
15. Tyssen R. Alcohol problems and depression: A 15-year longitudinal, nationwide study of mental health among Norwegian doctors. Project proposal, University of Oslo 2006.
16. Thomas N. Resident Burnout. JAMA 2004;292:2880-9.
17. Ackerley GD et al. Burnout among licensed psychologists. Professional Psychology: Research and Practice 1998;19:624-631.
18. Dyrbye LN, Thomas MR, Huschka MM et al. Personal Life Events and Medical Student Burnout: A Multicenter Study. Acad Med 2006;81:274-84.
19. Zoccolillo M, Murphy GE, Wetzel RD. Depression among medical students. J Affect Disord 1986;11:91-6.
20. Tyssen R, Vaglum P, Grønvold NT et al. The impact of job stress and working conditions on mental health problems among junior house officers. A nationwide Norwegian prospective cohort study. Med Educ 2000;34:374-84.
21. Dyrbye LN, Thomas MR, Shanafelt TD. Systematic review of depression, anxiety, and other indicators of psychological distress among U.S and Canadian medical students. Acad Med 2006;81:354-73.
22. Flanagan NA, Flanagan TJ. Satisfaction and job stress in correctional nurses. Res in Nurs and Health 2002;25:282-94.
23. Gallegos K, Bettinardi-Angres K, Talbott G. The effect of physician impairment on the family. Maryland Med J 1990;39:1001-7.
24. Tyssen R, Vaglum P, Grønvold NT et al. Suicidal ideation among medical students and young physicians: A nationwide and prospective study of prevalence and predictors. Journal of Affective Disorders 2001;64,69–79.
25. Richings JC, Khara GS, McDowell. Suicide in young doctors. Br J Psychiatry, 1986;149:475–8.
26. Spickard A, Gabbe S, Christensen J. Mid-career burnout in generalist and specialist physicians. JAMA 2002;288:1447–50.
27. Nelson NG, Dell'Oliver C, Koch C, et al. Stress, coping, and success among graduate students in clinical psychology. Psychol Rep 2001;88:759-67.
28. Fahrenkopf AM, Sectish TC, Barger LK. Rates of medication errors among depressed and burnt out residents: prospective cohort study. BMJ 2008;336:488-91.
29. Weiner EL, Swain GR, Wolf B et al. A qualitative study of physicians` own wellness-promotion practices. West J Med 2001;174:19-23.
30. Liberman N, Molden D, Idson LC, et al. Promotion and Prevention Focus on Alternative Hypotheses: Implications for Attributional Functions. J Pers Soc Psychol 2001; 80: 5-18.
31. Lockwood P, Jordan CH, Kunda Z. Motivation by positive or negative role models: Regulatory focus determines who will best inspire us. J Pers Soc Psychol 2002; 83:854-64.
32. Strauman TJ. Self-regulation and depression. Self and Identity 2002;1:151-7.
33. Wells A, Cartwright-Hatton S. A short form of the metacognitions questionnaire: properties of the MCQ-30.
34. Norcross, J. C (ed.) Psychotherapy relationships that work, Oxford University Press, New York 2002.
35. Hojat M, Mangione S, Nasca TJ et al.. An empirical study of decline in empathy in medical school. Medical Educ 2004;38:934-41.
36. Sherrard, Sarah. Empathy in psychology interns and medical residents: An investigation of the cognitive and emotional components of empathy. Dissertation Abstracts International: Section B: The Sciences and Engineering. 2007;68:1321.
37. West C, Huscka MM. Novotny PJ et, al. Association of Perceived Medical Errors with Resident Distress and Empathy: A Prospective Longitudinal Study. JAMA 2006;296:1071-8.
38. Baer RA, et al. Using Self-Report Assessment Methods to Explore Facets of Mindfulness. Assessment 2006;13:27-45.
39. Brown, K.W., & Ryan, R.M. The benefits of being present: Mindfulness and its role in psychological well-being. J Pers Soc Psychol 2003;84,822-848.
40. Epstein RM. Mindful Practice. JAMA 1999; 282: 833-9.
41. Germer, C.G., Siegel, R.D., & Fulton, P.R. (Eds.). Mindfulness and psychotherapy. New York: Guilford Press 2005.
42. Rosenzweig S et al. Mindfulness-based stress reduction lowers psychological distress in medical students. Teach Learn Med 2003;15:88-92.
43. Dyrbye LN, Thomas MR, Shanafelt TD. Medical student distress: causes, consequences, and proposed solutions. Mayo Clin Proc 2005;80:1613-22.
44. Shapiro SL, Schwartz GE, Bonner G. Effects of mindfulness-based stress reduction on medical and premedical students. Journal of Behavioral Medicine 1998;21:581-99.
45. Shapiro SL, Astin JA, Bishop SR et al. Mindfulness-Based Stress Reduction for Health Care Professionals: Results From a Randomized Trial. International Journal of Stress Management 2005;12:164–176.
46. Tacon AM, McComb J, Caldera Y, et al. Mindfulness meditation, anxiety reduction and heart disease: a pilot study. Fam Comm Health 2003;26:25-33.
47. Kimberly A. Coffey, MA. Mechanisms of Action in the Inverse Relationship Between Mindfulness and Psychological Distress. Complem Health Pract Rev 2008;Vol. 13, No. 2: 79-91.
48. Weissbecker I et al. Mindfulness-Based Stress Reduction and Sense of Coherence Among Women With Fibromyalgia. Journal of Clinical Psychology in Medical Settings 2002;9:297-307.
49. Eric L. Garland, MSW, LCSW. The Meaning of Mindfulness: A Second-Order Cybernetics of Stress, Metacognition, and Coping. Complementary Health Practice Review 2007;12,:15-30.
50. Kabat-Zinn, J. Full catastrophe living. Using the wisdom of your body and mind to face stress, pain, and illness. Bantam, New York 1990.
51. de Vibe M. Oppmerksomhetstrening – en metode for selvregulering av helse. Tidsskr No Lægeforen 2003;21:3062-4.
52. de Vibe M, Moum T. Oppmerksomhetstrening for pasienter med stress og kroniske sykdommer. Tidsskr No Legeforen 2006;15:1898-902.
53. Pradhan EK et al. Effect of MBSR in RA patients. Arthritis Rheum 2007;57:1134-42.
54. Nerdrum P, Rustøen T, Rønnestad MH. Student Psychological Distress: A psychometric study of Norwegian 1st year undergraduate students. Scand J of Educ Res 2006;50:95-109.
55. Tyssen R, Røsbak KM, Løvereide LK et al. Mental distress among medical student and university college students: the role of gender. 2009. To be submitted.
56. Tambs K, Moum T. How well can a few questionnaire items indicate anxiety and depression? Acta Psychiatr Scand 1993;87:364-7.
57. Strand BH, Dalgard OS, Tambs K et al. Measuring the mental health status of the Norwegian population: A comparison of the instruments SCL-25, SCL-10, SCL-5 and MHI-5 (SF-36). Nord J Psychiatry; 57(2):113-18.
58. Rø KEI, Gude T, Aasland OG. Does a self-referral program reach doctors in need of help? A comparison with the general Norwegian doctor workforce. BMC Public Health 2007;7:36 doi:10.1186/1471-2458-7-36.
59. Schaufeli WB, Martínez IM, Pinto AM, Salanova M, Bakker AB. Burnout and Engagement in University Students: A Cross-Cultural Study. Journal of Cross-Cultural Psychology 2002; 33; 464 -481. DOI: 10.1177/0022022102033005003.
60. Moum T, Naess S, Sorensen T et al. Hypertension labelling, life events and psychological wellbeing. Psychological Med 1990;20:635-46.
61. Røysamb E, Harris JR, Magnus P et al Subjective well-being. Sex-specific effects of genetic and environmental factors. Pers Individ Dif 2002;32:211-23.
62. Røysamb E et al. Happiness and Health: Environmental and Genetic Contributions to the Relationship Between Subjective Well-Being, Perceived Health, and Somatic Illness. J Personality and Soc Psychol 2003;85:1136-1146.
63. Krokstad S, Johnsen R, Westin S. Social determinants of disability pension: a 10-year follow-up of 62000 people in a Norwegian county population. Int J Epidemiol 2002;31:1183-91.
64. Vitaliano PP, Russo J, Carr JE et al. Medical-School Pressures and Their Relationship to Anxiety. J Nerv Men Dis 1984;172:730-6.
65. Holm M, Tyssen R, Stordal KI, et al. Self-development groups reduce medical school stress: a controlled intervention study. (resubmitted for publication 2009)
66. Hojat M, Gonnella JS, Nasca TJ et al. Physician empathy: Definition, Components, Measurements, and Relationships to Gender and Speciality. Am J Psychiatry 2002;159:1563-9.
67. Hojat M et al. Empathy Scores in Medical School and Ratings of Empathic Behaviour in Residency Training 3 years later. J of Soc Psychol 2005;145:663-71.
68. Baer RA. Construct Validity of the Five Facet Mindfulness Questionnaire in Meditating and Non-meditating Samples. Assessment 2008;15:329-342.
69. Torgersen S. Hereditary-environmental differentiation of general neurotic, obsessive, and impulsive hysterical personality traits. Acta Genet Med Gemellol (Roma) 1980; 29:193-207.
70. Torgersen S, Vollrath M. Personality types, personality traits, and risky health behaviour. In Vollrath M (ed) Handbook of personality and health. John Wiley, Chichester 2006; pp 215-233.
71. Røvik JO, Tyssen R, Gude T et al. Exploring the interplay between personality dimensions: A comparison of the topological and the dimensional approach in stress research. Pers Individ Diff 2007; 42:1255-66.
72. Vitaliano PP et al. The Ways of Coping Checklist – Revision and Psychometric properties- Multivariate Behav Res 1985;20:3-26.
73. Crowe E, Higgins E. Regulatory Focus and Strategic Inclinations: Promotion and Prevention in Decision-Making. Org Behav Hum Decision Proc 1997; 69: 117-132.
74. Kristeller JL, Hallett CB. An exploratory study of a meditation-based intervention for binge eating disorder. Journal of Health Psychology 1999; 4: 357-363.

**Appendix**

**Flow Diagram of the progress through the phases of the study (Oslo + Tromsø)**

# Forespørsel om deltakelse i forskningsprosjektet – Oslo (Invitation letter to the students)

**Oppmerksomhetstrening for å mestre stress og fremme personlig utvikling hos medisin- og psykologistudenter.**

# Bakgrunn og hensikt

Dette er et spørsmål til deg om å delta i en randomisert forskningsstudie for å undersøke om en metode for stressmestring kan redusere stress og fremme utvikling av empati og evne til tilstedeværelse hos medisin- og psykologistudenter. Studien foretas i et samarbeid mellom Nasjonalt kunnskapssenter for helsetjenesten og Det medisinske fakultet og psykologisk institutt, Universitetet i Oslo og Tromsø, og med støtte fra kvalitetsfondet i Den norske legeforening.

# Hva innebærer studien?

Du vil bli tilfeldig valgt ut til å få delta på et syv ukers kurs i stressmestring med ca 20-30 deltakere, eller være i kontrollgruppen som fortsetter studiet som vanlig. Kurset har seks ukentlige samlinger, utenom forelesningstid, på 1,5 time hver, i tillegg til en heldagssamling etter syv uker. Deltagerne vil få undervisning i enkle fysiske og mentale øvelser som fremmer avspenning og stressmestring, samt undervisning om stress og hvordan dette kan takles. Deltagerne oppfordres til å delta på alle samlingene og til å gjøre hjemmeøvelser som er anbefalt mellom samlingene. Kursmateriell i form av kurskompendium og CDer til hjemmeøvelser er gratis. Gjennom resten av studiet vil de som har deltatt på kurset få tilbud om 1,5 timers oppfølging i gruppe 2 ganger i året. De som deltar i kontrollgruppen, vil få tilbud om et tilsvarende kurs gratis etter studien er avsluttet.

# Mulige fordeler og ulemper

Metoden er tidligere prøvet ut i forsøk med studenter og har vist å kunne redusere stress og psykiske plager, øke velvære og evnen til empati og nærvær. Det er ikke rapportert om uheldige hendelser ved bruk av metoden.

Alle deltakerne vil svare på et spørreskjema ved 1) starten av studien, 2) etter kurset er holdt, 3) to år etter kurset, 4) før avsluttende eksamen, og 5) ett år etter avsluttet studium. Utover dette vil et tilfeldig utvalg av kursdeltagere, som ønsker det, inviteres til fokusgruppeintervjuer 3 måneder og 2 år etter kurset. Intervjuene vil ledes av en annen enn prosjektansvarlig. Intervjuene tas opp på lydbånd og transkriberes anonymt.

Alle deltakerne vil få betalt for utfylling av protokollene. De som er i intervensjonsgruppen vil motta totalt kr 500 etter utfylling av protokoll 1 og 2, og de som er i kontrollgruppen vil få totalt kr 700 etter utfylling av protokoll 1 og 2. Tilsvarende betaling vil bli gitt for ufylling av protokollene senere i studien. Betalingene skjer i form av gavekort på universitetsbokhandelen.

**Hva skjer med informasjonen om deg**

Informasjonen som registreres om deg skal kun brukes slik som beskrevet i hensikten med studien. Alle opplysningene vil bli behandlet uten navn og fødselsår eller andre direkte gjenkjennende opplysninger. En kode knytter deg til dine opplysninger gjennom en navneliste. Denne og transkriberte lydbånd vil bli slettet når studien er avsluttet i 2018-19. Det er kun autoriserte personer knyttet til prosjektet som har adgang til navnelisten og dataene fra undersøkelsen. Disse personene er underlagt taushetsplikt. Det vil ikke være mulig å identifisere deg i resultatene av studien når disse publiseres.

**Frivillig deltakelse**Det er frivillig å delta i studien. Du kan når som helst og uten å oppgi noen grunn trekke ditt samtykke til å delta i studien. Dette vil ikke få noen konsekvenser for deg videre. Dersom du ønsker å delta, samtykker du ved å krysse av samtykkeerklæringen på nettsiden [www.kunnskapssenteret.no/OT](http://www.kunnskapssenteret.no/OT) . Om du nå sier ja til å delta, kan du senere trekke tilbake ditt samtykke uten konsekvenser. Dersom du senere ønsker å trekke deg eller du har spørsmål til studien, kan du kontakte prosjektleder Michael de Vibe, tlf 91610957, [mfd@kunnskapssenteret.no](mailto:mfd@kunnskapssenteret.no) .

Professor Arild Bjørndal, Institutt for samfunnsmedisinske fag og førsteamanuensis Reidar Tyssen ved Avd. for atferdsfag, Institutt for medisinske basalfag, UIO, er medansvarlige for forskningsdelen av prosjektet.

# Kapittel A- utdypende forklaring av hva studien innebærer (Explanation of the study)

- **Kriterier for deltakelse:** Alle studenter som er i sitt andre semester på medisinstudiet eller 3. semester på profesjonsstudiet i psykologi høsten 2009, og våren 2010 vil bli invitert til å delta.

- **Bakgrunnsinformasjon om studien:** God pasientbehandling er avhengig av helsearbeidere som har utviklet personlige egenskaper for å kunne se, forstå og kommunisere godt med pasientene, i tillegg til å mestre stresset som jobben innebærer. Flere studier viser at studenter og helsepersonell sliter med betydelig stress, psykiske plager og lav livskvalitet, og at undervisning i stressmestring kan motvirke dette.
- **Metoden som prøves i studien:** Prosjektet vil prøve ut en anerkjent metode for stressmestring og personlig utvikling, Mindfulness-Based Stress Reduction (MBSR), eller oppmerksomhetstrening (OT) på norsk. Den består av et nøye beskrevet treningsprogram utviklet ved the University of Massachusetts Medical Center i 1979. I dag har mer enn 18000 pasienter og 4000 helsepersonell gått gjennom programmet der, og mer enn 250 helseinstitusjoner i USA og Europa tilbyr nå programmet. Det er god dokumentasjon på positive helsegevinster ved bruk av metoden.

Metoden består i å trene oppmerksomheten gjennom meditasjon, kroppsavspenning, yogaøvelser og oppmerksomhet på pusten i hverdagslivets stressende situasjoner. Metoden vektlegger full aksept av det man erfarer i kropp og sinn når man øver. Oppmerksomheten rettes så mot det som oppleves stressende for den enkelte og man utforsker nye måter å mestre dette på. Hver samling har et fastlagt innhold med tilhørende hjemmeøvelser. Elementer i programmet er: 1) Undervisning om oppmerksomhetstrening, stress, samspillet mellom kropp og sinn, kommunikasjon og mestring av stress. 2) Erfaringslæring gjennom øvelser i samlingene og hjemme med bruk av CD. 3) Gruppeprosess med refleksjon over hva deltakerne opplever når de øver.

- **Kontrollgruppen:** Fordi studien har til hensikt å undersøke om et kurs i stressmestring har noen vesentlig effekt for studentene, vil kontrollgruppen fortsette studiet som vanlig. Når studien er over, vil kontrollgruppen bli invitert til et tilsvarende kurs, arrangert over en helg, og uten kursavgift.
- **Forløp av studien:** Etter informasjon til studentene på starten av høstsemesteret 2009 og vårsemesteret 2010, får de som ønsker det utdelt informasjonsskriv og kan bli med i studien ved å registrere seg på internett på adressen [www.kunnskpssenteret.no/OT](http://www.kunnskpssenteret.no/OT) der de utfyller samtykkeerklæring og fyller ut startprotokollen. De vil også kunne maile eller ringe prosjektleder med spørsmål de måtte ha. Navnelisten med inkluderte studenter, vil bli administrert av en ansatt ved IT avdelingen på Kunnskapssenteret. To uker før kursene starter, vil alle som har fylt ut startprotokollen bli tilfeldig valgt ut til å delta på kurset eller til å være i kontrollgruppen, og de vil få beskjed om hvilken gruppe de kommer i via mail. Det vil bli to grupper for medisinstudenter og en gruppe for psykologstudenter høsten 2009 og det samme våren 2010. Disse vil bli lagt til ettermiddagen. Dersom man ikke kan møte på en av kurskveldene, kan man ta igjen denne møtekvelden på et av de andre kursene.

**Kapittel B - Personvern, økonomi og forsikring (Ethical issues)**

## Personvern

Opplysninger som registreres om deg er din alder, studie, sivilstatus og antall barn. Videre brukes spørreskjemaer for å kartlegge helse, livskvalitet, stress, stressmestring, empati, evne til tilstedeværelse, personlighet, viktige livshendelser og opplevelse av sosial støtte. Disse opplysningene innhentes 5 ganger i løpet av studien som går over 6 år.

Prosjektleder er ansatt ved nasjonalt kunnskapssenter for helsetjenesten, og ansvarlig for dataene er direktør Jon Arne Røttingen. Det er kun autorisert personell tilknyttet studien som vil ha tilgang til dataene og til listen som forbinder studentnavn med dataene. Disse personene er underlagt taushetsplikt. Denne listen vil slettes når studien er avsluttet. Intervjuene tas opp på bånd som transkriberes anonymt. Lydbåndene slettes ved prosjektslutt.

## Utlevering av materiale og opplysninger til andre

Ingen som ikke deltar i studien vil ha tilgang til dataene fra studien.

## Rett til innsyn og sletting av opplysninger om deg

Hvis du sier ja til å delta i studien, har du rett til å få innsyn i hvilke opplysninger som er registrert om deg. Du har videre rett til å få korrigert eventuelle feil i de opplysningene vi har registrert. Dersom du trekker deg fra studien, kan du kreve å få slettet innsamlede opplysninger, med mindre opplysningene allerede er inngått i analyser eller brukt i vitenskapelige publikasjoner.

**Økonomi**

Studien er finansiert gjennom forskningsmidler fra legeforeningens kvalitetsfond.

**Forsikring**

Da det ikke er rapportert om uheldige hendelser ved bruk av studiemetoden, tegnes det ikke noen forsikring for deltakerne i studien.

# Informasjon om utfallet av studien

Deltakerne vil få tilsendt nyhetsbrev med informasjon om studien.

**Samtykke til deltakelse i studien (Consent form)**

Jeg er villig til å delta i studien

----------------------------------------------------------------------------------------------------------------

(Signert av prosjektdeltaker, dato)

-----------------------------------------------------------------------------------------------------------------

Fullt navn med blokkbokstaver, og epostadresse.

Hvis jeg blir trukket ut til å delta på kurset, sier jeg meg villig til å kunne bli kontaktet for intervju i løpet av 1-3 måneder og 2 år etter at kurset er slutt. JA: NEI:

Jeg bekrefter å ha gitt informasjon om studien

----------------------------------------------------------------------------------------------------------------

(Signert, rolle i studien, dato)
