# Supplementary material for: Six-year positive effects of a mindfulness-based intervention on mindfulness, coping and well-being in medical and psychology students; Results from a randomized controlled trial
Source: PLoS One. 2018 Apr 24;13(4):e0196053. doi: 10.1371/journal.pone.0196053 (PMC5916495; doi:10.1371/journal.pone.0196053)
Supplement: S1 Text — (DOCX) [file pone.0196053.s002.docx]

**S 2 Principal Component Analysis of WCCL**

The suitability of data for factor analysis was assessed prior to conducting the factor analysis. Sample size adequacy was assessed by consulting the Kaiser-Meyer-Oklin (KMO) test of sampling adequacy. KMO was .828, exceeding the recommended value of .6 (Kaiser, 1974). All KMO values for individual items were >.503, which is just above the recommended limit of .5 (Field, 2009). Bartlett test of sphericity was significant (p=.000), supporting the factorability of the correlation matrix. An initial analysis was run to obtain eigenvalues for each component in the data. PCA revealed the presence of twelve components with eigenvalues exceeding 1. This solution was dropped because several components had only 1 item with strong factor loading. The scree-plot was then consulted as a criterion for factor extraction. The scree plot showed inflexions that justified retaining 3 components. When extracting 3 factors using oblimin rotation, items that make up the “problem focused coping” sub-scale in the original WCCL loaded most strongly on Component 1. Items that make up the three sub-scales of WCCL relevant to an avoidant style of coping (blame self, wishful thinking and avoidance) had strongest loading on Component 2. Items from the “seek social support” subscale of WCCL loaded most strongly on Component 3. 3 items from the avoidance sub-scales (item number 12, 35 and 38) relating to avoidance of social contact or hiding ones feelings from others, also loaded on component 3. These items were consequently considered part of the underlying construct represented by component 3 in further analysis, and the scoring was reversed. Item 18 (“Accepted the next best thing to what I wanted”) from the problem focused coping scale was deleted because of low factor loadings (<.04) . Item 27 (“Got mad at the people or things that caused the problem”), originally an item in the avoidance sub-scale, had a low and almost equal factor loading on all three components (.2-.3). As a consequence this item was not included in further analysis. The PCA was rerun after deleting items number 18 and 27. KMO statistics (KMO=.834) and Bartlett test of sphericity (.000) confirmed the data was still suitability for factor analysis. All KMO values for individual items were >.624 . All three components showed a number of strong loadings and most variables loaded substantially on only one component. According to Field (2009) a factor solution is stable if each factor has 10 or more loadings greater than 0.4 in a sample that exceeds 150. The three component factor solution for WCCL in this sample is very close to fulfilling this criterion as component 1, 2 and 3 had 10/14, 14/17 and 8/9 factor loading above .4, respectively. The correlations between factors were .210 (1 and 3), .077 (1 and 2) and .199 (2 and 3).
